# Periprosthetic Joint Infection: Biofilm Pathogenesis, Immune Dysregulation, and Emerging Prosthetic Interface Strategies

**DOI:** 10.3390/biology15131037

**Published:** 2026-06-29

**Authors:** Le Wan, Chan-Young Lee, Woo-Chul Jung, Youzhen Zheng, Kyung-Soon Park

**Affiliations:** Center for Joint Disease, Department of Orthopedic Surgery, Chonnam National University Medical School and Hospital, Hwasun-gun 58128, Republic of Korea; wle202302@gmail.com (L.W.); cnuhoslee@gmail.com (C.-Y.L.); 2617371@gmail.com (W.-C.J.); zyz941207@hotmail.com (Y.Z.)

**Keywords:** prosthesis-related infections, orthopedic implants, biofilms, antibacterial agents, osseointegration, biomaterials, immunomodulation

## Abstract

Periprosthetic joint infection (PJI) remains a major clinical challenge after total joint arthroplasty because of its association with prolonged antimicrobial therapy, repeated surgery, implant failure, functional disability, and substantial socioeconomic burden. Current strategies, including systemic antibiotics, debridement with implant retention, staged revision, and antibiotic-loaded cement spacers, remain indispensable but are limited by mature biofilm tolerance, protected microbial reservoirs, insufficient local drug penetration, persistent inflammation, and compromised periprosthetic bone repair. Increasing evidence indicates that PJI is not merely bacterial colonization of an implant surface, but a dynamic prosthetic interface disorder involving biofilm persistence, immune dysregulation, inflammatory osteolysis, and failed osseointegration. This review summarizes recent advances in anti-infective prosthetic interface design, emphasizing the transition from passive antibacterial coatings toward multifunctional immuno-antibacterial osseointegrative systems. The pathogenic basis of PJI is first discussed, including conditioning film formation, bacterial adhesion, biofilm maturation, protected reservoirs, immune evasion, and osteolysis. Current clinical management limitations are then evaluated, followed by emerging biomaterial strategies, including anti-adhesive and contact-killing surfaces, active antimicrobial coatings, mature biofilm disruption, biological antibiofilm therapies, smart infection-responsive delivery systems, and osteoimmunomodulatory interfaces. Particular attention is given to balancing early antibacterial activity with cytocompatibility, immune resolution, angiogenesis, mechanical durability, and long-term osseointegration. Finally, key translational barriers are highlighted, including load-bearing and tribological constraints, insufficiently standardized mature biofilm and animal models, limited clinical evidence for advanced smart materials, manufacturing reproducibility, sterilization compatibility, regulatory complexity, and application-specific clinical readiness. Future anti-PJI interfaces should evolve beyond unidirectional bacterial killing toward stage-specific systems integrating biofilm control, immune restoration, vascularized bone regeneration, and durable mechanical performance.

## Simple Summary

Infection around an artificial joint is one of the most serious complications after joint replacement surgery. It can cause long-term pain, repeated operations, implant failure, disability, and high medical costs. A major reason why these infections are difficult to cure is that bacteria can attach to the implant surface and form protective communities, called biofilms, which make them harder to remove with antibiotics or surgery alone. This review explains how infection develops at the surface where the implant meets the surrounding tissue, including bacterial attachment, biofilm formation, abnormal inflammation, bone loss, and poor implant fixation. It also summarizes new implant surface strategies that aim not only to kill bacteria, but also to prevent bacterial attachment, break down mature biofilms, control harmful inflammation, and support new bone growth. These next-generation approaches may help improve infection control, protect bone health, and increase the long-term success of joint replacement. By presenting current challenges and future directions, this review may help guide the development of safer and more effective artificial joint implants.

## 1. Introduction

Total joint arthroplasty (TJA) is among the most successful surgical interventions for restoring mobility, relieving pain, and improving quality of life in patients with end-stage joint disease. With population aging and expanding indications for arthroplasty, the global number of primary and revision joint replacement procedures continues to increase. However, periprosthetic joint infection (PJI) remains one of the most severe complications after arthroplasty, leading to prolonged antibiotic therapy, repeated surgical interventions, implant failure, functional disability, increased mortality, and substantial socioeconomic burden [1,2,3]. Epidemiological studies have reported that the incidence of PJI after TJA generally ranges from 1% to 3%, but even this relatively low incidence translates into a growing clinical and financial burden because of the rapidly increasing arthroplasty volume [1,4]. In a nationwide Korean database study, the annual incidence of PJI ranged from 2.3% to 2.8%, and the projected national cost of PJI treatment was expected to increase markedly by 2030 [1]. Recent clinical analyses further indicate that PJI is associated with significantly worse patient-reported outcomes and higher mortality compared with uncomplicated arthroplasty [2,3,5].

The clinical persistence of PJI is largely attributable to microbial biofilm formation on prosthetic materials, including titanium alloys, cobalt–chromium–molybdenum alloys, ultra-high-molecular-weight polyethylene, and polymethylmethacrylate (PMMA)-based bone cement [6]. Immediately after implantation, host proteins from blood and synovial fluid adsorb onto the biomaterial surface, forming a conditioning film that provides binding sites for bacterial adhesins. Staphylococci are the predominant pathogens in PJI. In a systematic review of 20 cohort studies including 3344 PJI cases, coagulase-negative staphylococci accounted for 31.2% of cases and *Staphylococcus aureus* for 28.8% [7]. These organisms can exploit adsorbed matrix proteins to initiate surface colonization and microcolony formation [6,8,9]. Non-staphylococcal pathogens, including Gram-negative organisms and fungi, are less frequent but clinically important in complex, recurrent, immunocompromised, or polymicrobial PJI and are therefore considered as pathogen-specific challenges in this review. Once established, bacterial communities produce an extracellular polymeric substance (EPS) matrix composed of polysaccharides, proteins, lipids, and extracellular DNA, thereby generating a highly structured biofilm architecture [6,10]. This biofilm matrix limits antibiotic penetration, reduces bacterial metabolic activity, promotes persister cell formation, and protects pathogens from host immune clearance [6,8,11]. As a result, bacteria embedded within mature biofilms can tolerate antimicrobial concentrations far exceeding those required to inhibit planktonic bacteria, making eradication by systemic antibiotics alone extremely difficult [11,12,13].

In addition to surface-associated biofilms, PJI pathogenesis involves deeper host–pathogen interactions within periprosthetic tissues and bone. Increasing evidence suggests that bacteria may persist within osteoblasts, intracellular niches, or bone-associated microenvironments, including the osteocyte lacuno-canalicular network, creating protected reservoirs that are difficult to eliminate through standard debridement or systemic antimicrobial therapy [6,11,14,15]. Moreover, the periprosthetic environment is shaped not only by infection but also by mechanical wear. Wear particles generated during long-term joint articulation can activate macrophages and foreign-body responses, amplify inflammatory signaling, and promote osteoclast-mediated bone resorption [16,17,18,19]. These wear-debris-driven inflammatory pathways can intersect with infection-associated immune responses, contributing to chronic inflammation, impaired bacterial clearance, periprosthetic osteolysis, and ultimately septic or aseptic loosening [16,17]. Therefore, PJI should not be viewed solely as a bacterial colonization event, but rather as a complex disorder of the prosthetic interface involving biofilm biology, immune dysregulation, inflammatory bone loss, and failed osseointegration [20].

Current clinical management of chronic PJI relies on surgical intervention combined with prolonged antimicrobial therapy. Debridement, antibiotics, and implant retention (DAIR) may be considered for selected acute infections, whereas one-stage or two-stage revision is commonly used for chronic or complex cases [21,22,23]. Although these strategies remain indispensable, their effectiveness is limited by mature biofilm tolerance, protected microbial reservoirs, insufficient antimicrobial penetration into poorly vascularized periprosthetic tissues, and difficulty in restoring a regenerative bone microenvironment. In particular, antibiotic-loaded PMMA spacers provide local antimicrobial delivery but do not actively support immune resolution, vascularized bone repair, or durable osseointegration [24,25].

These limitations have motivated a conceptual shift in PJI treatment from systemic antimicrobial rescue toward interface-centered therapeutic design. Because bacterial adhesion, biofilm maturation, immune activation, and bone remodeling converge at the prosthesis–tissue interface, this interface represents a critical therapeutic target. Next-generation anti-infective prosthetic systems are expected to move beyond passive antibiotic release and instead integrate multiple functions, including prevention of bacterial adhesion, disruption of mature biofilms, on-demand antimicrobial delivery, modulation of excessive inflammation, and support of osteogenesis and vascularized bone repair [26,27,28].

This review summarizes recent advances in anti-infective prosthetic interface design for PJI, focusing on the transition from conventional antibacterial strategies toward multifunctional systems that integrate biofilm control, infection-responsive therapy, immune regulation, and osseointegration. The review first outlines the biofilm–immune–bone mechanisms underlying PJI persistence, then discusses current clinical bottlenecks and emerging biomaterial strategies for stage-specific interface intervention. Key translational barriers related to mechanical durability, clinically relevant models, biosafety, manufacturing, and regulatory readiness are then highlighted. To improve conceptual clarity, the review is organized around two schematic frameworks: a stage-specific PJI progression timeline and an interface-centered model of next-generation anti-PJI prosthetic systems.

## 2. Pathogenesis of Periprosthetic Joint Infection

Periprosthetic joint infection (PJI) is not merely a localized proliferation of planktonic bacteria within the joint cavity. Instead, it represents a dynamic host–pathogen–biomaterial disorder that develops at the prosthetic interface [10,29]. Its pathogenesis involves rapid host protein adsorption, bacterial adhesion, biofilm maturation, antibiotic tolerance, immune evasion, and progressive destruction of the periprosthetic bone microenvironment. Recent evidence further indicates that bacterial persistence in orthopedic implant infections is reinforced by multiple protective niches, including mature biofilms, abscess-like structures, intracellular reservoirs, and invasion of the osteocyte lacuno-canalicular network (OLCN) [14,15,20,30]. Therefore, understanding PJI requires an integrated view of biofilm biology, local immune dysregulation, and impaired osseointegration.

### 2.1. Conditioning Film Formation and Initial Bacterial Adhesion

The earliest stage of PJI begins within minutes after prosthesis implantation. Once a metallic, polymeric, ceramic, or cement-based implant is introduced into the joint cavity, its surface is rapidly coated by host-derived proteins from blood and synovial fluid. These proteins, including fibrinogen, fibronectin, vitronectin, collagen, albumin, and immunoglobulins, form a conditioning film that changes the physicochemical properties of the implant surface and provides molecular ligands for both host cells and bacteria [31,32,33].

This process creates the classic “race for the surface,” in which host tissue integration and bacterial colonization compete for dominance at the newly implanted interface [34,35]. If osteogenic cells and immune defense mechanisms establish early control, stable osseointegration may proceed. In contrast, if bacteria occupy the surface first, irreversible adhesion and subsequent biofilm formation may occur [32,36].

Staphylococci are particularly adapted to this early colonization process. *Staphylococcus aureus* and coagulase-negative staphylococci, particularly *Staphylococcus epidermidis*, are among the most common PJI-related pathogens and express microbial surface components recognizing adhesive matrix molecules (MSCRAMMs) [37,38,39]. These adhesins bind to host matrix proteins deposited on the prosthetic surface and facilitate the transition from reversible adhesion to stable colonization [20]. Recent reviews have emphasized that *S. aureus* uses MSCRAMM-mediated adhesins to colonize implant surfaces and periprosthetic tissues, while regulatory systems such as accessory gene regulator and staphylococcal accessory regulator A coordinate adhesion, virulence, and biofilm adaptation [20,38,40]. This early adhesion phase is therefore not a passive attachment event, but an active and highly regulated host–pathogen interaction.

Surface material properties further influence bacterial adhesion. Titanium alloys, cobalt–chromium–molybdenum alloys, ultra-high-molecular-weight polyethylene, polyetheretherketone, and PMMA bone cement differ in roughness, hydrophobicity, charge, and protein adsorption behavior [33,41]. These surface-dependent differences can alter both bacterial attachment and host cell response. Thus, early PJI development is governed by the combined effects of bacterial adhesins, conditioning-film composition, implant surface chemistry, and the local synovial environment.

### 2.2. Biofilm Maturation, EPS Architecture, and Antibiotic Tolerance

After successful adhesion, bacteria proliferate and organize into microcolonies, which subsequently mature into structured biofilms. A mature biofilm is composed of bacterial cells embedded within an extracellular polymeric substance (EPS) matrix, consisting mainly of polysaccharides, proteins, lipids, and extracellular DNA (eDNA) [10,42,43]. This matrix provides mechanical stability, promotes intercellular communication, and protects embedded bacteria from antibiotics and immune cells [32,44].

The EPS matrix is a central determinant of PJI persistence. It functions as both a physical and biochemical barrier by restricting antimicrobial diffusion, neutralizing antimicrobial agents, reducing complement deposition, and limiting neutrophil migration [42,43,44,45,46]. In addition, the heterogeneous architecture of mature biofilms creates gradients of nutrients, oxygen, pH, and metabolic activity [43,47]. Bacteria in deeper biofilm layers often enter slow-growing or dormant states, making them less susceptible to antibiotics that target active cellular division [31,44].

Extracellular DNA is particularly important for biofilm structural integrity [44,48]. Yang et al. reported that eDNA acts as a critical scaffold within the EPS matrix, contributing to bacterial adhesion, matrix stabilization, metal ion chelation, metabolic coordination, antibiotic resistance, and horizontal gene transfer [44]. This explains why DNase or DNase-mimetic strategies can destabilize biofilms and enhance bacterial exposure to antimicrobial interventions. However, in established PJI, the presence of mature EPS architecture means that simple antimicrobial release may be insufficient unless the biofilm barrier itself is disrupted.

Clinically, biofilm maturation explains why systemic antibiotics frequently fail in chronic PJI. Magruder et al. noted that bacteria within biofilms exist in a slow-growing sessile state and are shielded from immune surveillance and antibiotic penetration, making biofilm presence a major driver of treatment failure and persistent infection [31]. Similarly, Akay and Yaghmur summarized that biofilm formation on orthopedic implants can increase antibiotic tolerance by 10- to 1000-fold compared with planktonic bacteria [10,32]. Therefore, effective PJI therapy must distinguish between prevention of early adhesion and eradication of mature biofilms, as these stages require different therapeutic strategies.

### 2.3. Pathogen-Specific Biofilm Diversity: Gram-Negative and Fungal PJI

Although staphylococci are the predominant pathogens in PJI and represent the major biofilm model in many implant-associated infection studies, non-staphylococcal pathogens also pose important clinical and translational challenges [49]. Gram-negative organisms, including *Pseudomonas aeruginosa*, may occur in complex, recurrent, healthcare-associated, or polymicrobial PJIs [50]. Compared with Gram-positive staphylococci, Gram-negative bacteria possess an outer membrane, periplasmic defense systems, efflux pumps, and distinct extracellular polymeric substance compositions, all of which may reduce susceptibility to conventional antibiotics and influence the performance of antimicrobial coatings or infection-responsive delivery systems [51].

Fungal PJIs are less common but clinically difficult to manage, particularly in immunocompromised patients or after repeated revision procedures [52]. *Candida species* can form yeast–hyphal biofilms with dense extracellular matrices that limit antifungal penetration, enhance tolerance to host immune clearance, and complicate implant salvage [53]. These pathogen-specific differences indicate that anti-PJI interface strategies should not be optimized exclusively using staphylococcal models. Future biomaterial studies should include Gram-negative, polymicrobial, and fungal biofilm models when clinically relevant, especially when evaluating broad-spectrum antimicrobial coatings, matrix-disruptive systems, or smart infection-responsive interfaces [50,51,52].

### 2.4. Hidden Bacterial Reservoirs and Deep Bone Persistence

Although prosthetic surface biofilms are central to PJI, bacteria may also persist in deeper protected niches. Recent evidence indicates that orthopedic implant infections are reinforced not only by surface biofilms but also by abscess-like structures, small-colony variants, intracellular persistence, and invasion of the osteocyte lacuno-canalicular network [14,20,30]. These reservoirs are clinically important because they may remain inaccessible to conventional irrigation, debridement, and systemic antibiotics.

*Staphylococcus aureus* is especially capable of establishing persistent bone-associated infection. It can invade osteoblasts, survive within intracellular compartments, and generate small-colony variants with reduced metabolic activity and enhanced antibiotic tolerance [20,54]. More importantly, *S. aureus* can invade the OLCN, a highly confined mineralized bone microstructure composed of osteocyte lacunae and submicron canaliculi. Because this network is deeply embedded in cortical bone and physically inaccessible to most immune cells, it may provide a protected reservoir for recurrent infection [15,20,30].

This concept is highly relevant for revision surgery. Even when infected prosthetic components are removed and the visible biofilm is debrided, bacteria embedded within periprosthetic bone may persist. These protected reservoirs can contribute to delayed recurrence after apparent infection control. Therefore, PJI should not be considered only a surface biofilm disease; it is also a bone-associated infection in which microbial survival within deep anatomical niches may compromise long-term treatment success.

### 2.5. Immune Evasion and Dysregulated Peri-Implant Inflammation

PJI persistence is also driven by immune dysfunction at the implant interface. Biofilms do not simply protect bacteria from antimicrobial agents; they actively reshape the local immune microenvironment. Zhang et al. described implant-associated biofilm infections as a combined result of bacterial biofilm resilience and immune dysregulation, in which biofilms suppress host immunity, impair tissue repair, and generate a permissive niche for persistent infection [55]. This immune dysfunction is characterized by ineffective bacterial clearance despite sustained inflammatory activation.

After implantation, the host immune system recognizes the prosthesis as a foreign body. Tissue injury, blood contact, and biomaterial exposure trigger protein adsorption, coagulation, neutrophil recruitment, monocyte infiltration, macrophage activation, and eventual foreign body reactions [55,56]. In the absence of infection, this inflammatory response should gradually resolve and transition toward tissue integration. However, bacterial contamination and biofilm formation can prevent immune resolution. Neutrophils may release reactive oxygen species, proteolytic enzymes, antimicrobial peptides, and neutrophil extracellular traps, but mature biofilms limit direct phagocytic access and reduce effective bacterial killing [55,57].

Macrophages are central regulators of the peri-implant immune response. Persistent exposure to bacterial products, biofilm-derived signals, and biomaterial-associated stimuli can drive macrophages into a dysfunctional state [55]. Instead of coordinating effective bacterial clearance followed by tissue repair, macrophages may remain in a dysfunctional state characterized by persistent inflammation, impaired bacterial clearance, and inadequate tissue repair. This state contributes to persistent cytokine release, impaired phagocytosis, immune exhaustion, and delayed tissue regeneration [20,55]. Therefore, immune evasion in PJI is not simply a failure of immune activation; rather, it reflects a maladaptive immune response that is simultaneously inflammatory, inefficient, and poorly regenerative.

### 2.6. Wear Debris, Foreign Body Reaction, and Periprosthetic Osteolysis

The PJI microenvironment is further complicated by mechanical wear and material degradation. During long-term joint articulation, prosthetic materials generate wear particles, including polyethylene, metal, ceramic, and cement debris. These particles are recognized by macrophages and other innate immune cells as danger-associated signals, inducing foreign body reactions, inflammasome activation, and chronic inflammatory cytokine production [18,58,59,60].

In aseptic loosening, wear debris-induced inflammation is a well-established mechanism of periprosthetic osteolysis. In PJI, however, wear particles may interact with pathogen-associated molecular patterns derived from bacteria and biofilms, thereby amplifying local immune dysregulation [18,61]. The combination of bacterial products and wear debris can sustain macrophage activation, increase pro-inflammatory cytokine production, and promote osteoclast-mediated bone resorption. This creates a pathological loop in which biofilm persistence, foreign body inflammation, and bone loss reinforce one another.

Inflammatory cytokines such as tumor necrosis factor-α, interleukin-1β, and interleukin-6 promote osteoclastogenesis in part through activation of the RANKL pathway [62,63]. At the same time, excessive inflammation and oxidative stress suppress osteoblast differentiation, angiogenesis, and mineralized matrix formation. The resulting imbalance between bone resorption and bone formation drives periprosthetic osteolysis, compromises osseointegration, and ultimately leads to septic loosening [28].

Together, infection-associated inflammation and wear debris converge on osteoclast activation, impaired osseointegration, and progressive implant instability [28].

### 2.7. Integrated Pathogenic Model: Biofilm–Immune–Bone Axis

**Figure 1:**
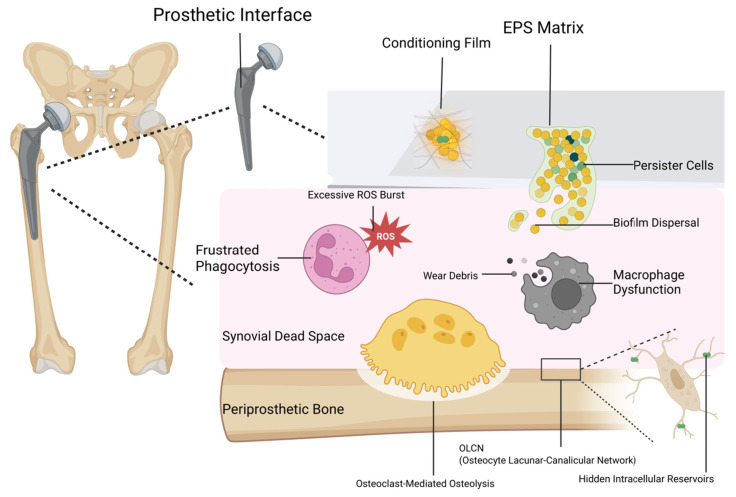
The biofilm–immune–bone axis in periprosthetic joint infection. Following total joint arthroplasty, host-derived proteins form a conditioning film on the prosthetic surface, promoting bacterial adhesion and EPS-rich biofilm development. Mature biofilms containing persister cells contribute to antimicrobial tolerance and immune evasion. In chronic PJI, bacteria may also persist in protected reservoirs, including intracellular niches and the osteocyte lacuno-canalicular network (OLCN). Biofilm-derived signals and wear debris further induce ROS production, macrophage dysfunction, osteoclast-mediated osteolysis, and impaired osseointegration. Created in BioRender. WAN, L. (2026) https://BioRender.com/7popirr (accessed on 20 May 2026).

**Figure 2:**
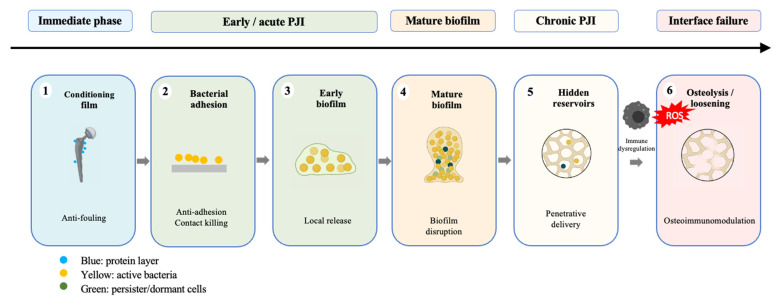
Simplified timeline of PJI progression and stage-matched intervention strategies. PJI progression at the prosthetic interface is summarized in six sequential but overlapping steps: (**1**) conditioning film formation after host protein adsorption, (**2**) bacterial adhesion to the conditioned implant surface, (**3**) early biofilm or microcolony formation, (**4**) EPS-rich mature biofilm development with active bacteria and persister/dormant cells, (**5**) bacterial persistence within hidden bone-associated reservoirs, and (**6**) chronic immune dysregulation with macrophage dysfunction and ROS production, leading to inflammatory osteolysis, failed osseointegration, and implant loosening. Corresponding stage-matched strategies include anti-fouling, anti-adhesion/contact killing, local antimicrobial release, biofilm disruption, penetrative delivery, and osteoimmunomodulation.

Collectively, PJI can be conceptualized as a biofilm–immune–bone axis. Host protein conditioning and bacterial adhesion initiate surface colonization, whereas EPS-rich biofilm maturation, protected microbial reservoirs, maladaptive inflammation, and osteoclast-mediated bone loss jointly sustain chronic infection and implant failure. This integrated model explains why bacterial killing alone is insufficient and supports therapeutic interfaces that combine biofilm prevention or disruption with immune regulation and bone regeneration [28,46,55]. The overall pathogenic framework is illustrated in Figure 1, while the temporal progression of PJI and corresponding stage-matched intervention strategies are summarized in Figure 2 and Table 1.

## 3. Current Clinical Management of PJI and Its Bottlenecks

Current management of periprosthetic joint infection (PJI) relies on a combination of systemic antimicrobial therapy, surgical debridement, implant retention or exchange, and local antibiotic delivery [64,65]. These approaches remain indispensable in clinical practice and have significantly improved infection control in selected patients. However, their efficacy is constrained by the biological properties of mature biofilms, limited drug access to the prosthetic interface, difficulty in defining the true extent of infected tissue intraoperatively, and the need to preserve sufficient bone and soft tissue for reconstruction. As a result, conventional treatment strategies often control but do not fully resolve the underlying biofilm–immune–bone pathology of chronic PJI [20,31,66].

### 3.1. Systemic Antibiotics and the Prosthetic Interface Penetration Barrier

Systemic antibiotics are central to perioperative prophylaxis and postoperative treatment of PJI [64,65]. In standard preventive protocols, systemic antimicrobial prophylaxis is administered near the time of surgical incision to achieve effective antimicrobial concentrations in serum and perioperative tissues during the contamination risk window. Although this strategy decreases the risk of early postoperative infection, it may not provide sufficient antimicrobial exposure at the implant surface, especially within ischemic or poorly vascularized postoperative tissues [36].

The major limitation of systemic therapy is that the prosthetic interface is not equivalent to a vascularized soft-tissue compartment. Once bacteria adhere to the implant surface and develop biofilms, they are embedded within an extracellular polymeric substance matrix that physically restricts drug diffusion and shields bacteria from host immune mechanisms. In addition, bacteria within mature biofilms frequently adopt slow-growing or dormant phenotypes, making them less susceptible to antibiotics that primarily target active bacterial replication [31]. These features explain why antibiotic concentrations effective against planktonic bacteria may be insufficient for biofilm-associated organisms. Recent reviews have emphasized that biofilm-associated bacteria can exhibit antibiotic tolerance up to approximately 1000-fold higher than planktonic counterparts, largely through physical sequestration, metabolic dormancy, and altered gene expression [20,31].

Therefore, systemic antibiotics alone are rarely adequate for chronic PJI with established biofilm formation [64,66]. Their role is essential but incomplete: they can suppress planktonic dissemination, reduce perioperative bacterial burden, and complement surgical intervention, but they cannot reliably eradicate mature biofilms or bacteria residing in protected bone-associated niches. For staphylococcal PJI, rifampicin-based combination therapy is clinically important because of its activity against biofilm-associated staphylococci, particularly in selected cases treated with DAIR or one-stage revision [67]. Rifampicin should not be used as monotherapy because of the risk of rapid resistance emergence, and its clinical use may be limited by drug–drug interactions. In this context, Leal et al. reported improved outcomes with rifampicin combination therapy in staphylococcal PJI treated with DAIR or one-stage revision and suggested that rifabutin may serve as a potential substitute when high-risk drug–drug interactions preclude rifampicin use [67]. Prolonged or repeated systemic antibiotic courses may also increase the risk of antimicrobial resistance, adverse drug reactions, and disruption of the host microbiome. These limitations have driven the development of local antibiotic delivery systems and implant surface modifications designed to increase antimicrobial activity directly at the prosthetic interface [36].

### 3.2. DAIR and Revision Surgery: Surgical Control Versus Tissue Preservation

Surgical intervention remains the cornerstone of PJI treatment. Depending on infection duration, pathogen virulence, host status, implant stability, and local bone and soft-tissue conditions, surgical options include debridement, antibiotics, and implant retention (DAIR), one-stage revision, two-stage revision, and, in selected cases, prolonged spacer retention or articulating spacer-based strategies [20,31,64,65].

The DAIR strategy is typically considered for acute postoperative or acute hematogenous infections when the implant remains stable and the biofilm is presumed to be immature [68,69]. Its advantage is preservation of the prosthesis and avoidance of major reconstructive surgery. Reported success rates for DAIR vary widely, but contemporary studies commonly report values of approximately 50–70% for early infections, depending on infection timing, joint type, follow-up duration, pathogen profile, and case selection [70]. Outcomes are generally better when DAIR is performed promptly, modular components are exchanged, the pathogen is susceptible to biofilm-active antibiotics, and host factors are favorable [68,69,71]. Once mature biofilm is established on retained components, DAIR becomes less reliable because mechanical debridement cannot consistently remove biofilm from all prosthetic surfaces, modular junctions, cement interfaces, or periprosthetic recesses [31,68].

For chronic PJI, two-stage revision remains the most widely used strategy in many clinical settings [65,72]. Infection eradication after two-stage revision is commonly reported at approximately 80–90% in many series, although published rates vary substantially depending on joint type, host factors, pathogen profile, surgical protocol, follow-up duration, and the definition of treatment success [73]. This approach usually involves removal of the infected prosthesis, extensive debridement, placement of an antibiotic-loaded spacer, systemic antibiotic therapy, and delayed reimplantation after infection control. Recent reviews note that two-stage revision remains a preferred treatment for chronic PJI in North America, but the prolonged treatment course is associated with substantial morbidity; some patients never reach reimplantation, and others experience significant functional decline during the interval period [31,72]. Similarly, recent analyses highlight that surgical decision-making during revision is limited by the difficulty of defining the true spatial extent of infection and biofilm burden: insufficient debridement may leave residual infection, whereas excessive debridement can compromise joint function and reconstructive potential [20]. Recent meta-analyses suggest that one-stage revision may achieve infection control comparable to two-stage revision in selected patients, but patient selection, pathogen profile, soft-tissue condition, and study heterogeneity remain major determinants of outcome [72,74].

Thus, the surgical dilemma in PJI is not simply whether to remove the implant, but how to balance infection eradication with preservation of viable bone, soft tissue, and future reconstructive capacity. In elderly patients or those with diabetes, immunosuppression, poor soft-tissue envelopes, osteoporosis, or major bone defects, repeated debridement and staged reconstruction may result in substantial bone loss and impaired biological fixation. This limitation is particularly important for next-generation biomaterials, because successful PJI management requires not only bacterial eradication but also restoration of a regenerative periprosthetic microenvironment.

### 3.3. Antibiotic-Loaded Bone Cement Spacers and Local Delivery Bottlenecks

Antibiotic-loaded bone cement (ALBC), most commonly based on polymethylmethacrylate (PMMA), is widely used during two-stage revision as a temporary spacer or as an adjunct for local antibiotic delivery. PMMA spacers provide several practical benefits: they maintain joint space, partially preserve soft-tissue tension, allow local delivery of antibiotics such as vancomycin, gentamicin, or tobramycin, and facilitate later reimplantation because of their moldability and mechanical stability [20,31,75,76].

However, PMMA-based delivery has important pharmacokinetic and biological limitations. Antibiotic release from cement spacers is typically characterized by an initial burst followed by a rapid decline and a prolonged low-level elution phase [75]. Although early high local concentrations may help suppress planktonic bacteria, later subtherapeutic concentrations may be inadequate for mature biofilm eradication and could contribute to selective pressure for resistant organisms [24]. Magruder et al. also emphasized that antibiotic-loaded bone cement provides only a limited elutable fraction of incorporated antibiotics, with an early high-release phase followed by variable and often declining elution depending on the antibiotic and formulation [31].

Another limitation is that PMMA is biologically inert and nondegradable. Once antibiotics have been depleted, the remaining cement may behave as a foreign material rather than an actively regenerative scaffold. Elsheikh et al. noted that after antibiotic release, PMMA may become an inert porous material that can potentially serve as a niche for bacterial colonization and infection [36]. In addition, PMMA spacers do not actively promote angiogenesis, osteogenesis, or immune resolution. This is particularly problematic in revision settings, where debridement often leaves compromised bone stock, devitalized tissue, and a poorly vascularized defect environment.

New local delivery strategies have therefore been proposed to overcome these limitations, including continuous intra-articular antibiotic irrigation, antibiotic-loaded ultra-high-molecular-weight polyethylene, degradable calcium sulfate carriers, hydrogels, and multifunctional biomaterial systems [25,28,31,36]. Nevertheless, many of these approaches remain in early clinical or preclinical stages, and their long-term safety, release kinetics, mechanical stability, and ability to support osseointegration require further validation.

### 3.4. Clinical Evidence for Antimicrobial Coatings and Remaining Uncertainty

Antimicrobial implant coatings have emerged as an interface-centered strategy to reduce bacterial adhesion and prevent biofilm formation at the primary site of colonization. These technologies include passive surface modifications that reduce bacterial adhesion, active surface modifications such as silver or iodine coatings, and perioperative local delivery systems such as defensive antibacterial coating hydrogel, gentamicin-loaded coatings/devices, or antibiotic-loaded calcium sulfate [36].

A recent network meta-analysis by Elsheikh et al. included 26 comparative studies involving 3592 patients and reported that coated implants were associated with lower infection rates than uncoated implants. In that analysis, defensive antibacterial coating hydrogel, gentamicin, iodine, and silver coatings significantly reduced infection risk, and coated implants were also associated with fewer postoperative complications, without a clear increase in operative time in the included studies [36]. These data suggest that antimicrobial coatings can provide clinically meaningful protection, particularly in high-risk procedures.

However, the same study also emphasized that clinical evidence remains incomplete. Head-to-head comparisons between coating technologies are limited, and available data are often derived from small or single-center studies [66]. Furthermore, coating selection must balance infection prevention with biocompatibility, cost, local toxicity, durability, and suitability for different anatomical and mechanical environments [36]. Therefore, while antimicrobial coatings represent an important advance beyond systemic prophylaxis and passive PMMA spacers, their clinical role remains dependent on procedure type, host risk profile, pathogen spectrum, and long-term implant performance.

The mechanistic categories of these coating strategies are discussed in the following section; here, the key point is that current clinical evidence remains strongest for relatively simple and clinically deployable antimicrobial platforms rather than highly complex smart biomaterial systems.

### 3.5. Paradigm Shift: The Prosthetic Interface as the Therapeutic Target

The limitations of systemic antibiotics, DAIR, staged revision, and PMMA-based local delivery converge on a central problem: conventional treatment does not sufficiently modify the prosthetic interface, where bacterial adhesion, biofilm maturation, immune activation, and bone remodeling occur simultaneously [36].

Accordingly, PJI management is shifting from reactive infection control toward proactive interface design. Rather than simply increasing antibiotic exposure, next-generation strategies aim to engineer implant surfaces and local delivery systems that prevent early colonization, disrupt established biofilms, regulate the inflammatory microenvironment, and support subsequent bone repair [20,66]. This rationale provides the basis for the following sections on biofilm-targeted surface engineering, mature biofilm disruption, infection-responsive delivery, and immunomodulatory osseointegration.

## 4. Biofilm-Targeted Prosthetic Surface Engineering

At the prosthetic interface, the earliest competition between host cell integration and bacterial colonization is often described as the “race for the surface” [32,34,77]. In the context of total joint arthroplasty (TJA), this competition is particularly important because bacterial adhesion to the prosthetic surface may rapidly progress to microcolony formation and biofilm maturation. Once a mature extracellular polymeric substance (EPS)-rich biofilm is established, eradication becomes substantially more difficult. Therefore, biofilm-targeted prosthetic surface engineering has emerged as an important preventive strategy to limit the transition from reversible bacterial attachment to irreversible biofilm-associated infection.

Surface engineering strategies can be broadly divided into three categories: anti-adhesive surfaces that reduce protein adsorption and bacterial attachment, contact-killing surfaces that destroy bacteria upon direct contact, and active antibacterial coatings that locally release antimicrobial agents or bioactive ions [32,77,78]. These approaches are conceptually attractive because they shift infection control from late-stage systemic rescue to early intervention at the primary site of bacterial colonization. However, their performance in PJI must be evaluated within the demanding environment of prosthetic joints, where synovial protein fouling, press-fit insertion, cyclic loading, micromotion, and long-term tribological wear may compromise coating integrity and biological function [58,77,78].

### 4.1. Anti-Adhesive Prosthetic Surfaces

Anti-adhesive surfaces aim to prevent the first step of biofilm formation by reducing nonspecific protein adsorption and bacterial attachment. Because the sterile implant surface is rapidly covered by plasma and synovial proteins after implantation, reducing nonspecific protein adsorption or modulating conditioning-film composition and conformation is a rational strategy to limit bacterial access to host-derived binding ligands. Polyethylene glycol, zwitterionic polymers, hydrophilic polymer brushes, and other anti-biofouling coatings have been explored to generate highly hydrated interfacial layers that resist nonspecific adsorption of proteins and microorganisms [32,77,79].

The underlying mechanism of anti-adhesive coatings is based on hydration-mediated steric repulsion and the reduction in favorable bacteria–surface interactions. Zwitterionic surfaces, for example, contain balanced positive and negative charges that strongly bind water molecules, forming a hydration shell that can reduce bacterial adhesion and biofilm initiation [79]. Similarly, PEGylated coatings can create a flexible hydrophilic barrier that limits protein adsorption and suppresses the ligand-mediated attachment of bacteria to the prosthetic substrate [32,77]. In orthopedic implant-associated infections, such passive bacterial-repelling strategies are particularly relevant during the perioperative period [32,77], when initial contamination and early surface colonization determine whether bacteria can establish a stable foothold at the implant interface [32,36].

However, anti-adhesive coatings are primarily preventive rather than therapeutic [32,78]. They are most effective before irreversible bacterial adhesion and biofilm maturation occur. Once microcolonies or mature EPS-rich biofilms have formed, purely anti-fouling surfaces are unlikely to eradicate infection. Therefore, anti-adhesive strategies are best viewed as early-stage preventive defenses rather than therapeutic solutions for established PJI.

### 4.2. Contact-Killing Prosthetic Surfaces

Contact-killing surfaces are designed to kill bacteria directly upon physical interaction with the implant surface. Unlike anti-adhesive coatings, which aim to prevent attachment, contact-killing interfaces require direct bacterial contact to disrupt membrane integrity or essential cellular functions. Common examples include quaternary ammonium compounds, cationic polymers, *N*-halamine-containing coatings, antimicrobial peptides, and nanostructured surfaces with mechano-bactericidal properties [32,55,77,80,81].

Cationic contact-killing surfaces typically exploit electrostatic interactions between positively charged surface groups and negatively charged bacterial membranes [32,77]. Upon contact, these interactions can disrupt membrane organization, induce pore formation, cause leakage of intracellular contents, and ultimately result in bacterial death. Antimicrobial peptide-functionalized surfaces operate through similar membrane-targeting mechanisms, although some peptides may also interfere with intracellular bacterial processes [80]. Compared with conventional antibiotic-releasing systems, these approaches may reduce systemic drug exposure and may be less dependent on bacterial metabolic activity.

Physical surface topographies also provide an antibiotic-independent approach to contact killing. Nanopillars, nanospikes, nanotubes, and other high-aspect-ratio micro/nanostructures can mechanically stretch or rupture bacterial membranes [81,82]. This mechano-bactericidal effect is attractive because it does not rely on antibiotic release and may reduce selection pressure for classical antibiotic resistance. In addition, certain nanoscale topographies may simultaneously regulate osteoblast adhesion, macrophage behavior, and mesenchymal stem cell differentiation, thereby offering a potential route toward dual antibacterial and osteogenic performance [81,83,84].

Nevertheless, contact-killing surfaces have important limitations. Their efficacy depends on direct bacterial contact, and they may be reduced when bacteria are shielded by conditioning films, fibrinous exudates, dead cell debris, or mature biofilm matrices [32,78]. In addition, accumulated host proteins and killed bacteria can mask active functional groups, thereby reducing long-term antibacterial activity. Thus, contact-killing surfaces are most relevant for early colonization control, whereas their durability in protein-rich synovial environments remains a major challenge.

### 4.3. Active Antibacterial Coatings and Local Antimicrobial Release

Active antibacterial coatings aim to deliver bactericidal agents directly at the implant interface. These systems include antibiotic-loaded coatings, metallic ion-based coatings, antimicrobial peptide delivery systems, and degradable local antimicrobial carriers [32,77,85]. Compared with systemic antibiotics, local release strategies can generate high antimicrobial concentrations near the prosthetic surface while reducing systemic exposure. Clinically, antimicrobial implant coatings such as defensive antibacterial coating (DAC) hydrogel, gentamicin-loaded coatings, iodine coatings, and silver coatings have shown protective effects in reducing orthopedic implant-associated infection risk [36].

Antibiotic-loaded coatings are among the most clinically intuitive strategies. They can deliver agents such as gentamicin, vancomycin, tobramycin, or rifampicin near the site of bacterial contamination. However, their efficacy is strongly influenced by release kinetics [75,85]. An initial burst may be beneficial for perioperative bacterial killing, but rapid depletion may leave the interface vulnerable during later stages of wound healing. Prolonged low-level elution may also create subtherapeutic concentrations that are insufficient for mature biofilm eradication and may contribute to antimicrobial selection pressure [24,36,75].

Metallic ion-based coatings represent another major class of active antibacterial surfaces. Silver, copper, zinc, gallium, and iodine-based systems can exert broad-spectrum antimicrobial effects through membrane damage, enzyme inhibition, oxidative stress induction, and disruption of bacterial metabolism [32,77,78]. Silver and iodine coatings are among the more clinically explored options, whereas copper- or zinc-based strategies are often designed to combine antibacterial activity with angiogenic or osteogenic effects [36,77]. A major challenge is achieving a therapeutic window in which bacterial killing is sufficient but cytotoxicity to osteoblasts, endothelial cells, and immune-regulatory cells remains limited [77,78].

Recent surface modification strategies increasingly aim to combine antibacterial activity with host cell compatibility [77,81,84]. This is clinically relevant because an implant surface that kills bacteria but impairs host tissue integration may still fail. Therefore, active coatings should be evaluated not only by bacterial reduction, but also by cytocompatibility, immune compatibility, and support for bone ingrowth.

### 4.4. Limitations in the Load-Bearing Prosthetic Joint Environment

Although anti-adhesive, contact-killing, and active-release coatings show encouraging antibacterial effects in vitro, their translation to PJI management is limited by the biological and mechanical complexity of prosthetic joints. Most coating studies are performed under static culture conditions using planktonic bacteria or early adhesion models, whereas clinical implants are exposed to synovial protein fouling, dynamic fluid shear, press-fit insertion, cyclic loading, micromotion, and long-term tribological wear [58,77,78]. These factors can alter coating durability, antimicrobial release, and antibacterial performance.

Mechanical stability is a major concern. Low-modulus polymeric or hydrogel-based coatings may be damaged, delaminated, or removed during press-fit implantation or long-term joint motion [77,85]. Protein adsorption, fibrin deposition, inflammatory exudates, and dead bacterial debris may also mask functional groups on contact-killing surfaces or reduce anti-adhesive performance [32,78]. For release-based systems, synovial dilution and biofilm sequestration may prevent sustained therapeutic concentrations at the infected interface.

Therefore, early surface engineering should be viewed primarily as a biofilm-preventive strategy rather than a definitive solution for chronic PJI [32,77,78]. In load-bearing arthroplasty, successful surface designs must retain antibacterial function despite protein fouling, synovial dilution, insertion-related damage, and long-term mechanical wear. These limitations provide the rationale for subsequent strategies that target established biofilms, infection-responsive delivery, and immune regulation, as discussed in the following sections [77,78,81]. Representative surface-engineering strategies for early biofilm prevention are summarized in Table 2.

## 5. Strategies for Mature Biofilm Disruption in PJI

Once periprosthetic joint infection (PJI) progresses into a delayed or chronic stage, bacteria are no longer present primarily as free planktonic organisms. Instead, they persist as highly organized biofilm communities on metallic components, polyethylene liners, cement interfaces, and periprosthetic tissues [86]. These mature biofilms are embedded within an extracellular polymeric substance (EPS) matrix composed of polysaccharides, proteins, lipids, and extracellular DNA (eDNA), which together provide structural stability and protection against antibiotics and immune attack [6,87]. In this setting, anti-adhesive or early contact-killing coatings are no longer sufficient. Therapeutic strategies must instead achieve biofilm-barrier penetration, matrix destabilization, bacterial killing, and immune-mediated clearance of residual pathogens [20,44,87].

Recent reviews have proposed that orthopedic implant infection management should shift from simple prevention toward “barrier breakthrough” strategies that can overcome biofilm-associated physical barriers, abscess-like structures, intracellular persistence, and bone-associated microbial reservoirs [20]. This concept is particularly relevant for chronic PJI, where bacteria may persist not only on the implant surface but also within periprosthetic bone microenvironments such as the osteocyte lacuno-canalicular network. Therefore, mature biofilm disruption should be understood as a multi-step process: degradation of the EPS matrix, exposure of protected bacteria, bactericidal intervention, and restoration of host immune access [28,55,87].

### 5.1. EPS Degradation and Enzymatic Matrix Disruption

The EPS matrix is a major determinant of biofilm tolerance [6,87]. It restricts antimicrobial diffusion, reduces complement deposition, limits neutrophil migration, and creates a protective niche in which bacteria can adopt slow-growing or dormant phenotypes [44]. Among EPS components, eDNA has attracted increasing attention because it functions as a structural scaffold that stabilizes biofilm architecture and promotes bacterial adhesion, metal ion binding, horizontal gene transfer, and antibiotic tolerance [44,88].

Enzymatic matrix degradation is therefore a rational strategy for weakening mature biofilms. DNase and DNase-mimetic systems can cleave eDNA, disrupt the biofilm scaffold, and increase bacterial exposure to antimicrobial agents [44,89,90]. Yang et al. developed an engineered nanoplatform combining Ce^4+^/nitrilotriacetic acid complexes as synthetic DNase mimics with gold nanorods as photothermal agents [44]. In this system, the DNase-mimetic component selectively degraded eDNA within the EPS matrix, destabilizing methicillin-resistant *Staphylococcus aureus* and *Pseudomonas aeruginosa* biofilms and facilitating deeper nanoplatform penetration. Subsequent near-infrared irradiation induced localized photothermal ablation, producing a synergistic antibiofilm effect [44].

This strategy illustrates an important design principle: mature biofilms should not be treated only by increasing bactericidal intensity; the matrix barrier itself must first be disrupted. However, native enzymes such as DNase may suffer from poor stability, high cost, and transient activity in vivo [90]. Synthetic nuclease mimics and catalytic nanoplatforms may partially overcome these limitations [44,89], but their long-term biosafety, clearance, catalytic selectivity, and performance in joint-like environments require further evaluation before translation to PJI treatment.

### 5.2. Physical Biofilm Disruption and Its Translational Constraints in Deep Joints

Physical approaches, including photothermal therapy, photodynamic therapy, magnetic hyperthermia, ultrasound-triggered therapy, and electromagnetic induction heating, have emerged as antibiotic-independent methods for disrupting mature biofilms [31,91,92]. These strategies are attractive because they may target metabolically less active or antibiotic-tolerant bacteria, reduce dependence on conventional antibiotics, and limit the selection pressure associated with prolonged antimicrobial exposure [31,55,92].

Photothermal therapy is one of the most widely studied physical approaches [44,93,94]. By converting external light energy into localized heat, photothermal agents can damage bacterial membranes, denature proteins, and disrupt biofilm integrity. In the Yang et al. nanoplatform, photothermal ablation was combined with DNase-mimetic EPS degradation to overcome the limited penetration of photothermal agents into mature biofilms [44]. This matrix-disruption-first strategy is particularly important because EPS can act as both a physical barrier and a thermal buffer [44,89], reducing the efficacy of photothermal killing when used alone. Light-assisted chemical approaches using redox-active or photosensitizing compounds have also been explored on implant-relevant titanium surfaces, but their application to deep prosthetic infections remains limited by tissue penetration, dose optimization, and host cell safety concerns [95,96].

Nevertheless, photothermal therapy faces important translational barriers in PJI [28,31]. Total hip and knee prostheses are located deep within muscle, capsule, synovium, and bone-adjacent tissues, making external light delivery to the infected implant interface challenging. Excessive localized heating may also damage synovial tissue, residual bone, neurovascular structures, or osteogenic cells. Therefore, while photothermal therapy is powerful in superficial wound or small-animal infection models, its direct application to deep prosthetic joint infections requires careful consideration of tissue penetration, temperature control, and implant geometry.

Electromagnetic induction heating may be particularly relevant for metallic joint implants because alternating magnetic fields can generate localized heating at conductive implant surfaces without requiring direct optical access. Magruder et al. highlighted electromagnetic induction heating as an emerging PJI technology that may disrupt biofilms on metallic implants, including surfaces inaccessible to conventional debridement [31]. However, this strategy also requires rigorous control of thermal dose to avoid collateral tissue injury, and its efficacy may differ across implant materials, geometries, and metal–polyethylene interfaces. Thus, physical biofilm disruption should be developed with joint-specific delivery constraints rather than extrapolated directly from superficial infection models [28,31].

### 5.3. Emerging Biological Therapeutics for Joint Cavity Delivery

Because physical ablation may be limited by penetration depth, thermal injury, or device complexity, biological and biochemical antibiofilm approaches have gained increasing interest for PJI. These strategies include D-amino acids, bacteriophage therapy, experimental antibody- or protein-based antibiofilm approaches, and agents that enhance antibiotic penetration or immune clearance [4,31,97,98].

D-amino acids are emerging experimental antibiofilm molecules that can interfere with bacterial cell wall remodeling and biofilm stability [4]. In the context of PJI, they have been explored as agents capable of promoting biofilm disassembly and improving the accessibility of embedded bacteria to antimicrobial treatment. Magruder et al. discussed D-amino acids as an emerging therapeutic approach that may enhance biofilm disruption, particularly when combined with other modalities such as photothermal therapy [4,31,93].

Bacteriophage therapy is another attractive strategy, especially for multidrug-resistant infections [97,98,99]. Phages can selectively infect and lyse target bacteria, and certain phages also produce depolymerases capable of degrading biofilm matrix components [97,98]. In principle, phage cocktails could be administered directly into the joint cavity during DAIR, loaded into hydrogels, or incorporated into revision spacers or porous implant materials for local delivery [97,99,100]. Their specificity may reduce off-target disruption of commensal flora and offer a personalized approach against resistant pathogens [31].

Despite these advantages, biological therapeutics face several challenges. Phage activity is strain-specific, requiring rapid pathogen identification and susceptibility testing [97,99,101]. Host immune responses may neutralize phages, while bacterial resistance to phages can emerge during therapy. Manufacturing, storage, quality control, and regulatory approval are also more complex than for conventional antibiotics [97,99,100]. Similarly, antibody-based or protein-based antibiofilm therapeutics must overcome issues of cost, stability, tissue penetration, and repeated-dose feasibility. Therefore, although biological therapies are promising, their role in PJI will likely depend on integration with surgical debridement, local delivery platforms, and standardized clinical protocols.

### 5.4. Combination Strategies for Mature Biofilm Disruption

Given the complexity of mature PJI biofilms, single-modality therapies are unlikely to be sufficient [28,55,87]. Effective management of mature biofilms generally requires coordinated disruption of the EPS matrix, killing or suppression of exposed bacteria, and restoration of immune clearance. This explains the growing interest in combination strategies that integrate enzymatic biofilm degradation, local antimicrobial release, physical activation, and immune modulation. Clinically available antiseptic combinations and irrigation-based strategies have been explored for biofilm control, but their effectiveness depends strongly on agent concentration, exposure time, combination design, and cytotoxicity or surgical compatibility [102,103,104].

One representative design principle is “matrix disruption followed by bacterial killing.” DNase-mimetic catalysis can weaken the eDNA scaffold, improving the penetration of photothermal agents, antibiotics, and metal ions while enhancing immune access to biofilm-associated bacteria [44,89]. Another principle is “local delivery with retention,” in which hydrogels, spacers, porous implants, or polymeric coatings maintain therapeutic agents in the joint cavity or periprosthetic defect area after debridement [28,31,93]. A third principle is “biofilm clearance with immune recovery,” in which biomaterials not only attack bacteria but also reduce excessive inflammation, improve macrophage function, and promote tissue repair [28,55].

For chronic PJI, combination therapy should be adapted to the surgical scenario. DAIR, two-stage revision, and reimplantation may require different delivery formats, ranging from injectable or irrigating systems to temporary spacers, degradable carriers, or durable anti-recolonization surfaces [28,31].

### 5.5. From Biofilm Disruption to Responsive Local Therapy

Although mature biofilm disruption strategies are promising, their clinical translation in PJI remains constrained by deep joint anatomy, polymicrobial biofilm heterogeneity, protected bone-associated reservoirs, and the inflammatory consequences of biofilm lysis [28,31,55]. Matrix degradation or physical disruption may expose embedded bacteria, but the released bacterial antigens, pathogen-associated molecular patterns, biofilm fragments, and inflammatory debris may also aggravate local inflammation if immune resolution is not simultaneously supported. These limitations have motivated the development of smart infection-responsive systems that localize antibiofilm activity to pathological microenvironments while reducing collateral damage to synovial tissue, residual bone, and osteogenic progenitors. The major strategies for disrupting established PJI biofilms are summarized in Table 3.

## 6. Smart Infection-Responsive Prosthetic Interfaces

The therapeutic objective in PJI management is shifting from continuous, nonspecific antimicrobial release toward infection-responsive precision therapy [55,105]. Conventional local delivery systems often release antimicrobial agents regardless of whether infection is present, which can lead to burst release, drug exhaustion, host cell toxicity, and selection pressure for antimicrobial resistance. In contrast, smart infection-responsive prosthetic interfaces or local delivery systems are designed to remain relatively inactive under physiological conditions and become activated only when exposed to infection-associated microenvironmental cues [105,106,107]. This approach may improve the spatial and temporal precision of antibiofilm therapy while reducing collateral damage to synovial tissues, residual bone, immune cells, and osteogenic progenitors.

In the context of PJI, smart responsive systems are particularly attractive because the infected joint cavity is pathologically distinct from a healthy prosthetic environment. Bacterial metabolism, neutrophil activation, biofilm formation, tissue injury, and foreign body inflammation collectively generate local signals that can be used as molecular triggers. These include acidic pH, elevated reactive oxygen species (ROS), bacterial or host-derived enzymes, altered redox balance, hypoxia, and externally applied stimuli such as light, ultrasound, magnetic fields, or heat [55,92,105,107]. By converting these pathological signals into therapeutic activation, responsive prosthetic interfaces aim to integrate antibacterial activity, biofilm disruption, immune regulation, and tissue repair [55,108].

### 6.1. Pathological Microenvironment Triggers in the Infected Joint Cavity

The infected periprosthetic environment is characterized by local microenvironments that differ markedly from physiological synovial and bone-healing conditions [55,105]. Bacterial metabolism can generate organic acids and lower the local pH, while inflammatory cells recruited to the implant interface release ROS, proteolytic enzymes, antimicrobial peptides, and inflammatory cytokines. Recent reviews have emphasized that after implantation, acute inflammation involves rapid recruitment of neutrophils, mast cells, and monocytes, with neutrophils releasing ROS and proteolytic enzymes to clear pathogens and tissue debris. If this response fails to resolve, persistent immune activation may transition into chronic inflammation and impaired tissue integration [55].

These pathological cues can serve as internal triggers for smart materials [105,106,107]. For example, acidic pH can open pH-sensitive gates, degrade acid-labile bonds, or alter the conformation and swelling behavior of pH-sensitive polymers. Elevated ROS can cleave ROS-sensitive linkages such as phenylborate ester or thioketal bonds, triggering drug release or hydrogel degradation [107,109,110]. Bacterial enzymes, including hyaluronidases, lipases, proteases, and nucleases, can be used to selectively degrade coatings or carriers within infected tissues. In addition, external stimuli can be applied to provide spatiotemporal control when endogenous signals are insufficient or heterogeneous.

For PJI, the key design challenge is selectivity. A responsive material should be activated by infection-related signals but remain stable during normal healing. It should also avoid excessive activation by sterile inflammation, mechanical wear debris, or postoperative tissue injury. This distinction is important because arthroplasty itself induces inflammation, protein adsorption, and immune cell recruitment even in the absence of infection. Therefore, next-generation responsive prosthetic interfaces should ideally integrate multiple triggers—such as pH plus ROS, bacterial enzyme plus inflammation, or external stimulus plus infection-specific targeting—to improve specificity and therapeutic precision [105,107,111].

### 6.2. pH-Responsive Prosthetic Interfaces

Acidification is one of the most widely used triggers in infection-responsive biomaterials. Local bacterial metabolism and inflammatory activity can reduce the pH of the infection microenvironment, enabling pH-sensitive coatings or carriers to release antimicrobial agents preferentially at infected sites. Recent reviews have summarized that bacterial infections may decrease local pH to approximately 5.5 in some infected microenvironments, which provides a rationale for pH-responsive biomaterials based on poly(methacrylic acid), silk fibroin, chitosan, metal–organic frameworks, and other pH-sensitive systems [83,106].

pH-responsive implant systems can be designed using swelling–deswelling polymer gates, acid-labile linkers, pH-sensitive charge conversion, or conformational changes in natural proteins [79,106,112]. For example, pH-sensitive molecular gates may remain closed under physiological pH and open under acidic infection-related conditions, allowing on-demand release of antimicrobial peptides or antibiotics [79,106]. This strategy reduces unnecessary elution in sterile conditions and may prolong antimicrobial availability during infection.

A representative example is the microenvironment-responsive PEEK implant reported by Chen et al., in which silk fibroin was used as a pH-responsive carrier for cerium oxide nanoparticles, while magnesium ions were immobilized through a polydopamine-mediated coordination network. Under acidic infection-related conditions, silk fibroin enabled the release of antibacterial cerium oxide nanoparticles, whereas magnesium ions were released more slowly to modulate macrophage phenotype and promote osteogenesis [113]. This design illustrates how pH-responsive systems can combine early infection-triggered bacterial killing with later osteoimmunomodulation, which is particularly relevant for implants that must achieve both infection control and osseointegration [112,113].

Nevertheless, pH-responsive approaches have limitations. The pH of infected tissues is often heterogeneous and may overlap with sterile inflammatory or ischemic microenvironments. In deep PJI, pH gradients within biofilms, synovial fluid, necrotic tissue, and periprosthetic bone defects may differ substantially. Therefore, pH responsiveness alone may not be sufficiently specific, and coupling pH sensitivity with other infection-associated triggers may improve precision [105,106,107].

### 6.3. ROS-Responsive Adaptive Systems

ROS are another important trigger in infected and inflamed tissues [107,108,109]. During PJI, neutrophils and macrophages may generate high levels of ROS in response to bacterial biofilms, wear particles, and necrotic tissue. Although ROS participate in antimicrobial defense, excessive ROS can damage synovial cells, osteoblasts, endothelial cells, and osteogenic progenitors, thereby impairing tissue repair [108,109]. Thus, ROS-responsive materials are attractive because they can both detect inflammatory oxidative stress and help restore redox balance.

Chen et al. developed a ROS-responsive adaptive injectable hydrogel for inflammatory mastoid bone repair. Although this model is not PJI-specific, the material design is highly relevant to infected bone defects. The hydrogel incorporated a ROS-sensitive dynamic phenylborate cross-linked network and catechol-containing components, allowing it to adapt to irregular tissue defects, exhibit tissue adhesion and self-healing behavior, release antibacterial TA@Ag nanoparticles, scavenge excess ROS, and regulate macrophage phenotype [109,110]. The authors reported effective antibacterial activity against *S. aureus* and *E. coli* in the reported model, along with concentration-dependent antioxidant activity and ROS-scavenging ability in macrophage assays [109,110]. This platform demonstrates how responsive hydrogels can integrate antimicrobial activity with oxidative-stress regulation and immune modulation [107,108,109].

For PJI management, ROS-responsive hydrogels may be especially useful as injectable adjuncts rather than load-bearing coatings. Their mechanical properties are generally insufficient for articulating surfaces, press-fit femoral stems, or high-shear bone–implant interfaces. However, they may be well suited for DAIR procedures, periprosthetic dead-space filling, debrided bone defect cavities, or the interval period between staged revisions. In these settings, a ROS-responsive hydrogel could provide local antibiofilm activity while reducing oxidative tissue injury and supporting immune resolution [108,109].

The translational value of ROS-responsive systems therefore depends on matching material mechanics to the clinical application. They should not be expected to replace hard prosthetic coatings on load-bearing surfaces, but they may complement surgery by filling irregular infected defects and delivering responsive antimicrobial and immunomodulatory therapy in non-articulating spaces.

### 6.4. Enzyme-/Multi-Stimuli-Responsive Systems and Gas-Releasing Platforms

Because PJI microenvironments are heterogeneous, single-trigger systems may not provide sufficient specificity or therapeutic control. Multi-stimuli-responsive systems are therefore increasingly being developed to integrate pH, ROS, enzyme activity, redox changes, and exogenous stimulation [105,107,111]. Such systems can function as “AND-gate-like” therapeutic platforms, in which drug release or activation occurs preferentially when multiple infection-associated cues are present.

Bacterial or inflammation-associated enzymes are particularly attractive triggers because they may provide greater biological specificity than pH or ROS alone. Enzyme-cleavable linkers can be designed to respond to bacterial proteases, lipases, hyaluronidases, or nucleases, enabling localized degradation of coatings or carriers within infected tissues [105,111,114]. In the joint cavity, hyaluronidase-responsive systems may be especially relevant because hyaluronic acid is a major component of synovial fluid and can be degraded during infection and inflammation. However, enzyme expression varies by pathogen, infection stage, and host response, so enzyme-responsive systems require careful validation in polymicrobial and clinically relevant PJI models [105,111].

Gas-releasing systems represent another emerging class of infection-responsive and immunomodulatory materials [55]. Nitric oxide, hydrogen sulfide, and carbon monoxide can exert antimicrobial, anti-inflammatory, vasoregulatory, and tissue-protective effects depending on dose, release flux, duration, and local concentration. Zhang et al. summarized that NO-, H_2_S-, and CO-releasing coatings can combine infection control with immune modulation and tissue regeneration, thereby mimicking endogenous defense pathways and reducing reliance on conventional antibiotics [55]. These platforms may be especially valuable when designed for controlled local release, because excessive or poorly controlled gas delivery may cause cytotoxicity or off-target effects.

Multi-stimuli strategies may also integrate endogenous triggers with external control. For example, pH/NIR dual-responsive coatings can release antibiofilm molecules under acidic infection conditions while using light-triggered phototherapy to enhance bacterial killing and promote osteogenic ion release [55,111]. Similarly, ultrasound- or magnetic-responsive systems may offer deeper external activation than optical approaches, whereas chemodynamic systems can exploit endogenous chemical reactions within infected microenvironments [92,115]. These multimodal platforms provide opportunities to combine biofilm disruption, antimicrobial activity, immune reprogramming, and tissue repair within a single therapeutic system.

For deep prosthetic infections, external triggers such as ultrasound or magnetic fields may provide better tissue penetration than light-based activation, but their use requires precise dosimetry, device compatibility, and careful safety validation [92].

### 6.5. Translational Positioning of Smart Responsive Interfaces in PJI

Smart responsive materials should be positioned according to the anatomical and mechanical requirements of PJI treatment. Soft injectable hydrogels, for example, are unlikely to be suitable as durable coatings on articulating surfaces or press-fit stems, where high shear, wear, and cyclic loading may cause coating damage [108,109]. Instead, they may be more appropriate for periprosthetic dead-space filling, local delivery after DAIR, treatment of irregular bone defects, or use during staged revision [108,109].

By contrast, hard or semi-rigid responsive coatings may be more suitable for non-articulating implant surfaces, porous titanium augments, PEEK cages, or revision components where surface modification can be protected from severe wear [112,113]. For load-bearing and articulating surfaces, responsive functions must be integrated without compromising tribological performance, corrosion resistance, or mechanical integrity. This requirement explains the growing interest in structure–function integrated designs rather than simply coating fragile responsive materials onto high-wear prosthetic surfaces [111,113].

The broader challenge is to align therapeutic activation with anatomical location, mechanical demand, and infection stage [55,111]. Therefore, smart infection-responsive interfaces should be evaluated not only by release behavior or antibacterial rate, but also by their ability to provide localized therapy without compromising immune recovery, bone regeneration, or mechanical suitability [55,108,113].

## 7. Immunomodulatory Osseointegration in PJI

Traditional anti-infective biomaterial design has largely focused on bacterial killing, antibiotic delivery, or inhibition of surface colonization. However, increasing evidence indicates that PJI is not only a disease of bacterial persistence, but also a disorder of the peri-implant immune microenvironment [55]. Mature biofilms, implant-derived wear particles, surgical trauma, and necrotic tissue can collectively generate a dysregulated inflammatory niche that impairs bacterial clearance, promotes osteoclast activation, suppresses osteogenesis, and compromises long-term osseointegration [16,18,28,55,116]. Therefore, the next generation of anti-infective prosthetic interfaces should not only enhance bacterial control but also restore immune homeostasis and support bone regeneration.

The concept of immunomodulatory osseointegration is based on the recognition that immune cells are active regulators of implant fate [116,117]. Neutrophils, macrophages, adaptive immune cells, osteoblast-lineage cells, osteoclasts, and endothelial cells interact dynamically at the prosthetic interface [18,116,117]. In an uncomplicated healing process, early inflammation should transition toward immune resolution, vascularization, osteogenic differentiation, and stable bone–implant integration. In PJI, this sequence is disrupted. Biofilm-protected bacteria and foreign-body signals can maintain persistent inflammation while simultaneously impairing effective antimicrobial defense. As a result, the peri-implant environment becomes both inflammatory and poorly regenerative.

Accordingly, immunomodulatory osseointegration aims to coordinate three therapeutic objectives: early bacterial clearance, timely inflammatory resolution, and late-stage vascularized bone regeneration [55,116,118]. This approach reframes host immunity from a passive background factor into a therapeutic target. Instead of maximizing antimicrobial potency at all times, materials should be designed to regulate immune responses in a stage-specific manner—supporting antimicrobial defense during early infection risk while preventing chronic inflammation and promoting osseointegration after bacterial burden is controlled.

### 7.1. Macrophage Polarization in Infected Periprosthetic Bone Repair

Macrophages are central regulators of the peri-implant immune microenvironment [116,117,118,119]. They participate in pathogen recognition, phagocytosis, cytokine secretion, foreign body responses, osteoclastogenesis, angiogenesis, and tissue repair. In the simplified M1/M2 framework, M1-like macrophages are associated with pro-inflammatory and antimicrobial functions, whereas M2-like macrophages are generally associated with inflammation resolution, tissue remodeling, angiogenesis, and pro-regenerative signaling. However, macrophage phenotypes in vivo are highly plastic and exist along a spectrum rather than in binary states [116,118]. Therefore, for implant design, the key objective is not to force a fixed M1 or M2 state, but to guide a timely transition from controlled antimicrobial inflammation toward pro-resolving and pro-regenerative immune activity [116,118,120].

In early infection, controlled pro-inflammatory macrophage activation can be beneficial because it enhances phagocytosis, reactive species generation, and antimicrobial cytokine production. However, persistent macrophage activation can be detrimental. Long-term production of TNF-α, IL-1β, IL-6, and other inflammatory mediators suppresses osteoblast differentiation, promotes osteoclastogenesis, and contributes to periprosthetic osteolysis [16,18,62]. Thus, the success of PJI revision depends not only on bacterial eradication, but also on preventing prolonged inflammatory polarization that damages the residual bone bed.

Sequential immunomodulation has therefore become an attractive design principle. Zhou et al. constructed a multilayer MgO-modified PEEK surface capable of spontaneous dynamic regulation of the ionic and immune microenvironment [121]. In the early stage, the outer MgO layer rapidly degraded, generating a local alkaline environment and releasing a relatively high concentration of Mg ions. This early microenvironment disrupted bacterial energy metabolism and promoted controlled M1-like macrophage activation, thereby supporting bacterial clearance. In the later stage, the inner MgO layer degraded more slowly and sustained Mg ion release, promoting osteoblast proliferation and differentiation while inducing M2-like macrophage polarization. In a rat femoral implantation model, this sequential immune-enhanced strategy improved both antibacterial activity and osseointegration [121].

This study provides a representative example of stage-specific immune design [118,121]. Its significance lies not only in the use of MgO, but also in the broader concept that the implant surface can be engineered to change its biological function over time. For PJI, such temporal coordination is critical: early after implantation or revision, the interface must suppress residual bacteria; later, it must reduce inflammation and support bone repair. This dynamic logic is more consistent with the biological sequence of infection control and bone healing than a static coating that releases a single antibacterial agent at a fixed rate.

### 7.2. Neutrophils, ROS, NETs, and Inflammatory Injury Resolution

Neutrophils are among the earliest immune cells recruited to infected prosthetic joints and provide essential antimicrobial defense through phagocytosis, degranulation, ROS generation, antimicrobial peptide release, and formation of neutrophil extracellular traps (NETs) [122,123,124]. In acute infection, these responses help restrict bacterial dissemination. However, in mature biofilm-associated PJI, neutrophil function may become frustrated because bacteria embedded within EPS matrices are difficult to engulf or eliminate [55].

Frustrated neutrophil activation can produce excessive ROS, proteases, inflammatory mediators, and NETs, which may interact with biofilm-associated inflammation in chronic infections [122,125]. Although these factors contribute to antimicrobial defense, they can also damage synovial tissue, osteoblast-lineage cells, endothelial cells, and residual bone. Persistent oxidative stress may further amplify macrophage activation and impair the transition toward tissue repair. Therefore, controlling excessive neutrophil-driven oxidative inflammation is important for restoring a regenerative periprosthetic environment. Recent reviews emphasize that neutrophils are not merely short-lived antimicrobial cells, but versatile regulators of inflammation, tissue injury, immune signaling, and disease progression [126].

ROS-responsive and antioxidant biomaterials provide one strategy to address this problem. Chen et al. developed a ROS-responsive adaptive injectable hydrogel containing a dynamic phenylborate cross-linked network and TA@Ag nanoparticles [109]. Although developed for inflammatory mastoid bone repair rather than PJI, the design is highly relevant to infected bone defects because it combines antibacterial activity, ROS scavenging, tissue adhesion, self-healing properties, and macrophage phenotype regulation [109]. In the context of PJI, such materials may be particularly useful as injectable adjuncts after DAIR or during staged revision, where irregular bone defects and dead spaces require both antimicrobial protection and inflammatory microenvironment control [55,109].

For prosthetic joint applications, the key translational issue is proper positioning. ROS-responsive hydrogels are unlikely to withstand high-wear articulating surfaces or press-fit insertion sites. However, they may be useful in non-load-bearing periprosthetic cavities, dead spaces, and debrided bone defects. In these settings, reducing excessive ROS may help protect osteogenic progenitors, support macrophage transition toward reparative phenotypes, and create conditions more favorable for vascularized bone repair.

### 7.3. Osteoimmune Crosstalk, Osteoclastogenesis, and Periprosthetic Osteolysis

PJI-associated bone loss results from a disruption of osteoimmune balance [16,18,116]. During chronic infection, persistent bacterial antigens, biofilm debris, wear particles, and inflammatory cytokines activate immune pathways that favor osteoclastogenesis and inhibit osteogenesis. Pro-inflammatory mediators such as TNF-α, IL-1β, and IL-6 can promote RANKL signaling, disturb the RANKL/osteoprotegerin balance, and increase osteoclast differentiation and bone resorption [18,62,127]. Meanwhile, inflammatory and oxidative stress suppress osteoblast maturation, mineralization, and bone matrix deposition.

Wear particles further amplify this process. Periprosthetic osteolysis has traditionally been viewed as a macrophage-driven foreign-body inflammatory response to wear debris, but recent studies show that the inflammatory cell infiltrate around loosened implants is heterogeneous and involves multiple innate and adaptive immune populations [18]. When biofilm-derived pathogen-associated molecular patterns coexist with wear-derived danger signals, osteoimmune dysregulation becomes more severe [16,113]. This may help explain why PJI can lead to septic loosening and compromised revision outcomes, particularly when residual inflammation and bone loss persist despite apparent infection control.

The RANKL/OPG axis is particularly relevant to implant loosening. A recent clinical study in periprosthetic osteolysis after total ankle arthroplasty reported that osteolysis was associated with increased osteoclastogenesis through an elevated RANKL/OPG ratio in synovial fluid [127]. Although this study was performed in ankle arthroplasty and not specifically in PJI, it is consistent with the broader concept that inflammatory synovial environments can drive osteoclast activation and periprosthetic bone loss. For PJI, targeting osteoclastogenic inflammation may therefore complement antibacterial therapy by protecting the bone bed required for reimplantation and osseointegration [18,62,116].

Thus, immunomodulatory osseointegration should include not only macrophage phenotype control but also regulation of osteoclast activity, osteoblast function, angiogenesis, and inflammatory cytokine networks. Materials that reduce excessive NF-κB signaling, promote IL-10 and pro-regenerative cytokines, or restore the RANKL/OPG balance may help interrupt the cycle of infection-associated bone destruction [62,113,116].

### 7.4. Molecular Mechanisms of Osteoimmunomodulation at the Prosthetic Interface

At the molecular level, several inflammatory and osteogenic pathways connect immune regulation with osseointegration. NF-κB is one of the most important signaling hubs because it regulates inflammatory cytokine production, macrophage activation, and osteoclast differentiation [62,113]. In PJI, excessive NF-κB activation may sustain chronic inflammation and promote RANKL-mediated osteolysis. Therefore, controlled attenuation of excessive NF-κB signaling after bacterial burden is reduced may be beneficial for immune resolution and bone regeneration.

Chen et al. designed a microenvironment-responsive PEEK implant coated with silk fibroin, cerium oxide nanoparticles, and immobilized Mg ions [113]. Under acidic infection-related conditions, the silk fibroin-based system enabled antibacterial release of cerium oxide nanoparticles, while Mg ions were slowly released to regulate macrophage phenotype and enhance osteogenesis. Bioinformatics and experimental analyses suggested that modulation of the NF-κB signaling pathway contributed to the anti-inflammatory effects. The Mg-mediated immunomodulatory effect promoted M2-like macrophage polarization and increased the production of pro-regenerative factors such as IL-10, VEGF, and BMP-2, thereby improving osseointegration [113,116].

This design demonstrates an important principle: infection-responsive antibacterial activity and osteoimmunomodulation can be integrated within a single prosthetic surface. Early antibacterial effects help reduce pathogen burden, while later Mg-mediated immune regulation supports inflammation resolution, angiogenesis, and osteogenic differentiation. Importantly, the material does not rely solely on direct stimulation of osteoblasts; instead, it uses immune cells as intermediaries to create a regenerative microenvironment [113,116,117]. This indirect immune-mediated enhancement may be especially relevant in PJI revision, where residual bone tissue is inflamed, vascularity is compromised, and direct osteogenic signaling alone may be insufficient.

Other ions and bioactive components may also contribute to osteoimmunomodulation. Magnesium is associated with macrophage regulation and osteogenic support, copper can promote angiogenesis through HIF-1α/VEGF-related pathways, zinc can support osteogenesis and antibacterial defense within an appropriate dose range, and strontium has been widely explored for osteogenic and anti-resorptive effects [116,118,128]. However, these ions also require careful dose control because excessive concentrations may become cytotoxic or pro-inflammatory. Therefore, the central challenge is not simply choosing a bioactive ion but controlling its spatial and temporal release to match the transition from infection control to tissue regeneration.

### 7.5. From Static Immunomodulation to Stage-Specific Immune Programming

Many conventional immunomodulatory biomaterials aim to reduce inflammation or promote M2-like macrophage polarization. However, in infected prosthetic environments, excessive early immunosuppression could be counterproductive because bacterial clearance still requires antimicrobial immune activation. Conversely, prolonged pro-inflammatory activation causes tissue injury and osteolysis. Therefore, PJI requires stage-specific immune programming rather than static immunomodulation [55,118,121].

In a practical design framework, the interface response can be divided into three overlapping phases [118,121]. In the early antibacterial phase, the interface should support controlled innate immune activation, bacterial phagocytosis, and biofilm suppression. In the immune-resolution phase, the interface should limit excessive ROS, reduce inflammatory cytokines, and prevent chronic synovitis or foreign-body inflammation. In the regenerative phase, the interface should promote vascularization, osteoblast differentiation, bone matrix formation, and stable osseointegration. The MgO-modified PEEK multilayer system provides one example of this temporal strategy by using differential MgO degradation to generate early antibacterial immune activation followed by later reparative immune polarization [121].

This stage-specific concept is particularly important for revision arthroplasty [16,55]. After debridement, the local environment often contains residual bacterial burden, inflammatory debris, poor vascularity, and compromised bone stock. A material that only kills bacteria may leave behind a damaged, poorly regenerative bone bed. Conversely, a material that only promotes osteogenesis may fail if residual biofilm persists. Therefore, next-generation immuno-antibacterial prosthetic interfaces should be designed to coordinate bacterial clearance, inflammatory resolution, angiogenesis, and osteogenesis in a time-dependent manner [116,118,121].

### 7.6. Translational Considerations for Immunomodulatory Osseointegration

Although immunomodulatory osseointegration is conceptually attractive, its translation requires careful biological validation. First, the M1/M2 framework is useful but oversimplified [116,118]; macrophage phenotypes in human PJI are heterogeneous and influenced by pathogen species, biofilm maturity, wear debris, host comorbidities, and local oxygenation [16,55,118]. Future studies should therefore combine conventional marker analysis with broader immune profiling, cytokine network assessment, and functional assays of phagocytosis, inflammatory resolution, and tissue repair.

Second, immune-regulatory materials should be evaluated beyond bacterial counts and bone volume. Relevant endpoints should include neutrophil activity, ROS levels, macrophage phenotype transition, osteoclastogenesis, vascularization, osteoblast differentiation, and long-term fixation [28,116,117]. Finally, immunomodulation must be stage-specific: excessive early immune suppression may permit bacterial persistence, whereas prolonged immune activation may worsen tissue injury and osteolysis. The therapeutic window for immune modulation must therefore be defined according to infection stage, bacterial burden, implant location, and host immune status [116,118].

Overall, immunomodulatory osseointegration reframes infection control and bone regeneration as interconnected immune-mediated processes. The most promising prosthetic interfaces will be those that dynamically coordinate antimicrobial activity, immune resolution, angiogenesis, and bone integration [55,116,118]. Representative infection-responsive and osteoimmunomodulatory interface strategies are summarized in Table 4.

## 8. Balancing Antibacterial Activity and Osseointegration in PJI Management

The major challenge in anti-infective prosthetic design is no longer limited to maximizing bacterial killing [28,129]. In PJI management, the more difficult task is to coordinate biofilm control, inflammatory resolution, vascularized bone regeneration, and durable osseointegration within a mechanically demanding joint environment. Conventional antibacterial modifications often pursue high local antimicrobial potency through concentrated antibiotics, metallic ions, reactive oxygen species, or membrane-disruptive surfaces. Although these approaches may reduce bacterial burden, excessive antimicrobial intensity can also impair osteoblast viability, endothelial function, macrophage homeostasis, and the regenerative capacity of the residual bone bed. Therefore, next-generation prosthetic interfaces must achieve an antibacterial–osseointegrative balance rather than unidirectional sterilization [28,129,130,131].

This balance is particularly important in revision arthroplasty. Extensive debridement, chronic inflammation, poor vascularity, and bone loss create a compromised periprosthetic microenvironment in which infection control and bone repair must occur simultaneously. A surface that kills bacteria but prevents osteoblast attachment or induces persistent inflammation may still fail through poor biological fixation. Conversely, a surface designed only for osteogenesis may be vulnerable to recolonization if residual biofilm or dormant bacteria persist. Thus, the optimal anti-PJI interface or material system should provide strong early antibacterial activity, avoid prolonged cytotoxicity, support immune resolution, and promote late-stage osseointegration [28,130,132].

### 8.1. Antibacterial Potency Versus Cytocompatibility at the Prosthetic Interface

A persistent dilemma in antibacterial implant design is the trade-off between antimicrobial potency and host cell compatibility. Metallic ions such as silver, copper, zinc, and gallium can exert broad-spectrum antibacterial effects through membrane disruption, enzyme inhibition, oxidative stress, and interference with bacterial metabolism [131,133,134,135,136]. However, these effects are often dose dependent. When local ion concentrations exceed the tolerance of host cells, they may induce mitochondrial dysfunction, oxidative damage, impaired osteoblast differentiation, delayed endothelial repair, or excessive inflammatory activation.

Silver-containing biomaterials are a representative example [137,138,139]. Silver is highly effective against a broad range of bacteria and has been widely explored for hard-tissue infection prevention, but its biological performance depends strongly on release kinetics, dose, coating stability, and local tissue exposure. Recent work on silver-coated bone and implant materials emphasizes that silver must be delivered within a therapeutic window that preserves bone formation while maintaining antibacterial activity [137,138,140,141]. Similarly, copper provides antibacterial and angiogenic potential, but excessive copper release may become cytotoxic or pro-oxidative [133,134,142]. Therefore, the key issue is not whether metallic ions are antibacterial, but whether their release can be controlled in a way that decouples bacterial killing from host tissue damage.

To address this “toxicity window,” recent strategies increasingly combine antibacterial agents with bioactive matrix components, coordination networks, or mineral phases that regulate ion release and improve tissue compatibility. For example, hydroxyapatite-reinforced CoCrMo-3Cu alloys integrate copper-mediated antibacterial activity with hydroxyapatite-mediated tribofilm formation [58]. In this design, copper contributes to intrinsic antibacterial activity, while hydroxyapatite improves wear resistance and reduces cobalt ion release by forming a solid lubricating tribofilm under load-bearing conditions [58]. This approach is highly relevant to arthroplasty because it integrates antibacterial function into a mechanically robust alloy rather than relying on fragile surface coatings.

Another strategy is to use natural polymers or protein-based matrices to constrain ion release. In the microenvironment-responsive PEEK implant developed by Chen et al., silk fibroin and polydopamine-based coordination were used to regulate the release of cerium oxide nanoparticles and magnesium ions [113]. This design enabled infection-triggered antibacterial activity under acidic conditions while allowing sustained Mg^2+^-mediated immunomodulation and osteogenic support [113]. Such systems illustrate a broader principle: the therapeutic effect of bioactive ions depends not only on their identity, but also on the surrounding delivery matrix, release kinetics, and interaction with immune and osteogenic cells.

### 8.2. Surface Nanotopography for Dual Antibacterial and Osteogenic Functions

Surface nanotopography offers an antibiotic-independent strategy to influence both bacterial and host cell behavior [81,143]. Certain nanopillars, nanospikes, nanotubes, nanogrooves, and hierarchical micro/nano structures can reduce bacterial attachment or viability by modulating bacterial–surface interactions and, in some cases, imposing mechanical stress on bacterial membranes [81,82,144]. This mechano-bactericidal effect is attractive because it does not rely on antibiotic release and may reduce classical resistance selection pressure. At the same time, nanoscale topography can modulate protein adsorption, integrin clustering, focal adhesion formation, cytoskeletal organization, and mechanotransduction in osteogenic cells.

The osteogenic effects of nanotopography are mediated in part through cell–matrix adhesion and mechanosensitive signaling. Recent reviews of titanium alloy surface topography highlight that micro/nano features can regulate osteogenic cells, endothelial cells, and macrophages [81,143], thereby influencing osseointegration through both direct cellular adhesion and immune-related pathways [84]. Specific nanoscale features can enhance integrin-mediated focal adhesion kinase activation and downstream mechanotransduction pathways associated with osteogenic differentiation and matrix mineralization.

Recent experimental evidence supports the feasibility of multifunctional micro/nano-textured titanium surfaces. Ziegelmeyer et al. reported that micro/nano-textured titanium demonstrated bactericidal, osteogenic, angiogenic, and anti-inflammatory properties, suggesting that mechano-bactericidal topographies may simultaneously reduce infection risk and support osseointegration [81]. This is particularly important because the ideal PJI interface should not simply repel bacteria but should also actively promote host tissue integration.

However, translating bactericidal nanotopographies into arthroplasty remains challenging. Sharp nanostructures may be damaged during press-fit insertion, plastic deformation, or long-term micromotion. If these features detach or collapse, antibacterial function may be lost, and wear-like debris may aggravate inflammation. Therefore, the most clinically plausible approach may be hierarchical micro/nano design. In such systems, microscale porous structures provide mechanical protection and bone ingrowth space, while nanoscale features are integrated within protected pore walls or non-articulating surfaces [145]. For revision implants, this design could be particularly useful in porous titanium augments or metaphyseal cones, where protected internal surfaces can support both bacterial control and bone ingrowth [146].

### 8.3. Osteogenic and Angiogenic Functionalization in Post-Debridement Dead Space

After surgical debridement for chronic PJI, the local environment often contains devitalized bone, inflammatory synovium, impaired microcirculation, and irregular dead space. In this context, stable osseointegration cannot be achieved by osteogenic stimulation alone. Bone repair requires coordinated angiogenesis–osteogenesis coupling, because vascular invasion provides oxygen, nutrients, immune cell trafficking, and osteoprogenitor recruitment. HIF-1α is a central regulator of this process and is involved in vascular invasion, osteoprogenitor differentiation, and bone regeneration [147].

Bioactive ions can be used to reactivate angiogenesis–osteogenesis coupling in compromised bone defects. Copper is particularly relevant because it can support antimicrobial activity and stimulate angiogenic signaling. Copper-containing biomaterials have been reported to promote osteogenesis–angiogenesis coupling, partly through HIF-1α/VEGF-related pathways [133,134,147,148,149,150]. In the PJI setting, this dual role is valuable because the same material platform may contribute to bacterial control and vascularized repair.

Nevertheless, angiogenic functionalization must be carefully balanced. Excessive or uncontrolled copper release can be cytotoxic, whereas insufficient release may fail to provide antimicrobial or vascular benefits. Therefore, delivery systems based on coordination networks, bioactive ceramics, hydrogels, or degradable mineral phases may be necessary to maintain copper or other pro-angiogenic ions within a safe and effective range [133,142,151]. In addition, angiogenic cues should be integrated with immunomodulatory signals. Pro-regenerative macrophage responses can support vascularization and osteogenic repair through factors such as VEGF, IL-10, and BMP-2-related signaling, providing an immune-mediated route to tissue repair [28,113,121]. Thus, the most effective post-debridement materials may be those that coordinate antibacterial activity, macrophage reprogramming, angiogenesis, and osteogenic differentiation.

In practical terms, osteogenic and angiogenic functionalization may be especially important in dead-space fillers, porous revision implants, metaphyseal augments, bone defect scaffolds, and injectable hydrogels used after debridement [28,151]. These locations are less exposed to articulating wear than joint bearing surfaces but are critical for biological fixation and long-term revision success.

### 8.4. Stage-Specific Design: Temporal Coordination of Antibacterial Defense and Bone Regeneration

The time course of infection recurrence and bone healing creates conflicting requirements for PJI biomaterials. In the immediate postoperative or post-revision period, residual planktonic bacteria and early adherent bacteria should be rapidly reduced or controlled. During the subsequent healing phase, excessive inflammation and cytotoxic antimicrobial exposure should decline to allow vascularization, osteoblast differentiation, and bone ingrowth. Therefore, stage-specific design has emerged as a central principle for anti-PJI prosthetic interfaces [28,113,121].

The multilayer MgO-modified PEEK system developed by Zhou et al. provides a representative example of this temporal logic [121]. In the early stage, rapid degradation of the outer MgO layer generated an alkaline microenvironment and released Mg ions, disrupting bacterial energy metabolism and inducing controlled M1-like macrophage activation to support bacterial clearance. In the later stage, slower release from the inner MgO layer promoted osteoblast proliferation and differentiation and induced M2-like macrophage polarization, thereby supporting osseointegration [121]. This design demonstrates that dynamic modulation of the ionic and immune microenvironment can synchronize antibacterial activity with tissue integration.

Similarly, the microenvironment-responsive PEEK implant reported by Chen et al. integrates pH-triggered antibacterial activity with Mg^2+^-mediated osteoimmunomodulation. Under acidic infection-related conditions, antibacterial cerium oxide nanoparticles are released, while Mg^2+^ provides slower immunomodulatory and osteogenic effects through pathways including attenuation of excessive NF-κB signaling and macrophage phenotype regulation [113]. Together, these studies, together with recent sequential antibacterial–immunomodulatory titanium implant designs, suggest that stage-specific interface design may be more biologically appropriate than static antimicrobial release [28,113,121,152].

### 8.5. Mechanical and Tribological Compatibility as Part of Biological Balance

For PJI management, antibacterial–osseointegrative balance must also include mechanical and tribological compatibility. A coating that performs well in static antibacterial assays may fail clinically if it delaminates during press-fit insertion [77,78], generates debris during cyclic loading, or alters the wear behavior of articulating surfaces. The mechanical environment of total hip and knee arthroplasty is therefore not a secondary engineering concern; it directly shapes the biological fate of the prosthetic interface.

This issue is especially important for soft coatings, hydrogels, and fragile nanostructures. These materials may be highly effective in vitro but unsuitable for high-shear or articulating surfaces. In contrast, load-bearing alloys or integrated surface architectures may provide more durable solutions [58,78]. The CoCrMo-3Cu-HA alloy developed by Bandyopadhyay et al. illustrates this direction by embedding antibacterial copper and wear-modulating hydroxyapatite into a load-bearing alloy [58]. The hydroxyapatite phase contributed to tribofilm formation, improved wear resistance, and reduced cobalt ion release, while copper provided antibacterial activity [58]. This structure–function integration is highly relevant for arthroplasty because it addresses infection prevention, wear safety, and material durability simultaneously.

Thus, anti-PJI interface design should be matched to anatomical loading conditions. Articulating surfaces require wear resistance and minimal debris generation, whereas press-fit stems, cups, porous augments, and bone-contacting surfaces require coating adhesion, shear resistance, osteoconductivity, and bacterial resistance. Softer injectable or responsive materials are better suited for dead-space filling, local delivery, or irregular bone defects than for high-wear load-bearing surfaces [28,58,77,78].

### 8.6. Design Principles for Antibacterial–Osseointegrative Interfaces

Several design principles emerge from current evidence [28,78,129]. First, antibacterial activity should be strong during the early infection risk period but should avoid prolonged cytotoxic exposure. Second, antimicrobial agents and bioactive ions should be released within a therapeutic window that preserves osteoblast, endothelial cell, and immune cell function [131,133,135,138]. Third, surface structure should support both bacterial control and host integration, preferably through protected hierarchical micro/nano architectures. Fourth, immune regulation should be stage-specific rather than broadly anti-inflammatory. Fifth, mechanical durability and tribological safety should be treated as part of biological performance rather than as separate engineering concerns [58,77,78].

**Figure 3:**
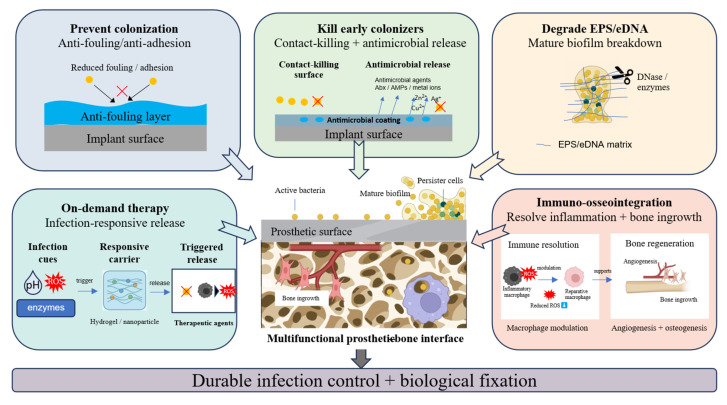
Next-generation anti-PJI prosthetic interface integrating antibacterial, biofilm-disruptive, infection-responsive, and immuno-osseointegrative functions. The schematic summarizes complementary strategies acting at the prosthetic–bone interface, including prevention of early colonization, killing of early colonizers, EPS/eDNA matrix degradation, infection-responsive therapeutic release, and immune–bone regeneration. Together, these functions aim to achieve durable infection control and biological fixation.

Thus, the ideal anti-PJI prosthetic interface should be dynamically adaptive rather than defined by maximal bacterial killing alone. It should integrate early colonization control, mature biofilm disruption, infection-responsive therapy, immune resolution, and long-term osseointegration [28,113,121]. This interface-centered design concept is illustrated in Figure 3.

## 9. Translational Challenges and Clinical Readiness in PJI Management

Despite rapid progress in nanotechnology, infection-responsive delivery systems, and immunomodulatory prosthetic interfaces, most advanced or emerging anti-PJI biomaterials remain confined to in vitro studies or small-animal models [25,28]. A substantial translational gap persists between promising laboratory performance and real-world application in total joint arthroplasty (TJA) or revision surgery [25,28,153]. This gap reflects the complexity of PJI itself: mature biofilms, polymicrobial infection, compromised host immunity, synovial fluid protein fouling, poor vascularity, bone loss, and high mechanical loading all coexist within the prosthetic joint environment.

Therefore, clinical readiness should not be judged solely by antibacterial rate or biofilm reduction in simplified assays [28,153]. A clinically viable anti-PJI interface or material system must demonstrate durable antimicrobial performance, compatibility with osseointegration, mechanical stability under implantation and joint loading, long-term biosafety, manufacturing reproducibility, sterilization compatibility, and efficacy in clinically relevant infection models.

### 9.1. Clinical Evidence Versus Preclinical Performance

A major translational reality is that many sophisticated antibacterial or stimuli-responsive materials have not entered routine clinical use. In contrast, technologies with relatively simple mechanisms and established safety profiles—such as antibiotic-loaded cement, silver coatings, iodine coatings, gentamicin-loaded coatings, and defensive antibacterial coating hydrogels—currently have the most direct clinical evidence [36,154,155,156,157].

The recent network meta-analysis by Elsheikh et al. included 26 comparative human studies involving 3592 patients and evaluated antimicrobial implant coatings for preventing orthopedic implant-associated infections [36]. The analysis reported that DAC hydrogel, gentamicin-loaded coatings/devices, iodine coatings, and silver coatings were associated with lower infection risk than uncoated implants in the included studies, with coated implants also associated with fewer postoperative complications and no increase in operative time [36]. This finding suggests that clinically established coating systems can provide meaningful protection, especially in high-risk procedures.

However, this evidence also highlights the current limitation of advanced biomaterials. Many pH-responsive, ROS-responsive, enzyme-responsive, photothermal, or immune-programming platforms show impressive preclinical performance, but their clinical value remains uncertain because few have been tested in human arthroplasty settings [25,28,158]. The transition from experimental efficacy to clinical adoption requires more than biological novelty. It requires safety, durability, scalability, compatibility with surgical workflows, and clear superiority over existing clinical options.

Several clinical or near-clinical coating technologies illustrate this uneven translation. Iodine-supported titanium implants have been evaluated clinically for infection prevention or infection control support in compromised hosts and high-risk reconstruction settings, while newer iodine-based titanium coatings are being explored for acute periprosthetic infection mitigation [64,159,160]. Gentamicin-coated intramedullary nails have shown encouraging results in open tibial fractures and other high-risk fracture settings [155,156,161,162]. Silver-coated implants, particularly in tumor endoprostheses and revision reconstruction, have also been investigated [155,163], with systematic reviews suggesting potential infection prevention benefits, although effect sizes, indications, and long-term safety remain under evaluation [155,164].

Thus, the current clinical landscape suggests that the most translatable anti-infective strategies are not necessarily the most biologically complex. Instead, they are those that combine adequate antibacterial function with acceptable safety, mechanical robustness, predictable manufacturing, and practical surgical use [25,158,165].

### 9.2. Mechanical Durability and Tribological Challenges in Arthroplasty

Mechanical durability is a defining requirement for anti-PJI prosthetic interfaces [58,78,158]. Cementless femoral stems, acetabular cups, tibial components, and revision augments may experience substantial shear forces during press-fit insertion. After implantation, hip and knee prostheses are exposed to cyclic loading, micromotion, synovial lubrication, corrosion, and long-term wear. These mechanical and tribological conditions are rarely reproduced in standard antibacterial assays [28,158,166].

This limitation is particularly important for polymeric coatings, hydrogels, peptide coatings, and fragile nanostructures. Although these materials may reduce bacterial adhesion or kill bacteria in static culture, they may delaminate, abrade, crack, or lose function during surgical insertion or long-term joint motion. Detached coating fragments may also behave as wear debris, potentially promoting foreign-body inflammation and periprosthetic osteolysis. Therefore, antibacterial function must be evaluated together with coating adhesion, wear resistance, corrosion behavior, fatigue stability, and debris biocompatibility [58,78,158].

The hydroxyapatite-reinforced CoCrMo-3Cu alloy developed by Bandyopadhyay et al. provides a useful example of a mechanically integrated strategy [58]. Instead of applying a fragile antimicrobial coating, the authors incorporated copper and hydroxyapatite into a load-bearing CoCrMo alloy fabricated by laser-based directed energy deposition. Copper provided intrinsic antibacterial activity, whereas hydroxyapatite contributed to tribofilm formation, improved wear resistance, and reduced cobalt ion release under tribocorrosion conditions [58].

This type of structure–function integration is highly relevant to arthroplasty because it recognizes that infection control at arthroplasty interfaces cannot be separated from tribological performance [58,158]. For articulating or high-load regions, antimicrobial activity must not compromise wear performance, corrosion resistance, or mechanical strength. Conversely, soft responsive materials may be better suited for non-load-bearing applications, such as dead-space filling, local delivery after DAIR, or periprosthetic bone defect repair.

### 9.3. Limitations of Preclinical Models: The Need for Standardized PJI Modeling

Another major barrier to translation is the limited clinical relevance of many preclinical models [28,167,168]. Numerous studies rely on planktonic bacterial inoculation, early adhesion assays, subcutaneous implants, small rodent bone defects, or non-load-bearing infection models [28,167,168]. These systems are useful for mechanism screening but do not fully reproduce the clinical features of PJI, including mature biofilm formation, synovial fluid exposure, closed joint cavities, prosthetic components, host comorbidities, polymicrobial infection, and mechanical loading.

The systematic review by Zhang et al. evaluated functional biomaterials for PJI treatment in preclinical animal studies and included only studies using validated animal models with prosthetic joint components [28]. The review found that functional biomaterials demonstrated promising mechanisms, including localized antimicrobial delivery, biofilm disruption, tissue regeneration, and immunomodulation. However, the authors emphasized persistent limitations in model standardization, mechanistic validation, and translational feasibility [28].

This has important implications for future research. Materials intended for PJI treatment should be tested against mature biofilms on protein-conditioned implant-relevant surfaces rather than planktonic bacteria alone [28,167,168]. Animal models should incorporate prosthetic components, clinically relevant pathogens, appropriate bacterial loads, joint-like anatomy, and, when feasible, mechanical loading. Models should also reflect clinically important host factors such as aging, diabetes, osteoporosis, immune compromise, and poor soft-tissue conditions.

Large-animal models may be particularly important for evaluating clinically realistic implants and load-bearing performance [168,169,170]. Sheep, goats, pigs, and canine models can better approximate human joint size, bone quality, implant geometry, and surgical handling compared with rodents. However, large-animal studies are expensive, technically demanding, and require standardized protocols. Therefore, a tiered evaluation strategy may be most practical: in vitro mature biofilm and immune assays for early screening, small-animal models for mechanistic validation, and large-animal load-bearing PJI models for late-stage translational testing [28,167,168].

### 9.4. Manufacturing, Sterilization, and Regulatory Considerations

Clinical translation also depends on manufacturing and regulatory feasibility [165,171]. Many advanced biomaterials are difficult to produce reproducibly at scale. Multilayer coatings, nanostructured surfaces, stimulus-responsive hydrogels, phage-loaded systems, and ion release platforms require strict control over composition, coating thickness, drug loading, release kinetics, surface roughness, degradation rate, and batch-to-batch consistency [165,172].

Sterilization is another important issue [165]. Gamma irradiation, ethylene oxide, autoclaving, plasma sterilization, and aseptic processing may alter polymer networks, degrade peptides or proteins, change nanoparticle oxidation states, reduce phage activity, or modify drug release behavior. Therefore, sterilization compatibility should be evaluated early rather than after biological efficacy has already been demonstrated. Shelf-life stability is also essential, especially for commercial implant coatings or ready-to-use intraoperative delivery systems.

Regulatory pathways may be complex because many anti-PJI technologies combine devices, drugs, biologics, and active delivery functions [165,171]. Antibiotic-loaded coatings, metal ion-releasing implants, bacteriophage-loaded hydrogels, and immune-modulating surfaces may be classified differently depending on jurisdiction and primary mode of action. This increases the need for robust preclinical evidence, toxicology testing, release profile characterization, and long-term safety evaluation.

Cost and manufacturing scalability will also influence clinical adoption. Relatively simple antimicrobial coatings or clinically established local delivery systems may be more readily scalable, whereas complex multilayered, nanoparticle-loaded, or cell-instructive smart interfaces may face higher production costs, batch-to-batch variability, sterilization challenges, and more demanding regulatory pathways [171,172]. Therefore, future clinical trials should not only assess infection rates but also evaluate revision risk, functional recovery, complications, quality of life, cost effectiveness, and long-term implant survival [36,154,164].

### 9.5. Clinical Readiness by Application Scenario

Not all anti-PJI biomaterials should be expected to serve the same clinical role. Their translational potential depends strongly on the intended application site and mechanical environment.

For primary arthroplasty prevention, durable antimicrobial coatings on non-articulating surfaces may be useful, especially in high-risk patients [36,64,154]. Silver, iodine, gentamicin-loaded coatings/devices, and DAC hydrogel have some of the strongest clinical evidence among currently used or clinically evaluated antimicrobial coating-related strategies [36,154,155,156]. Iodine-supported titanium implants may also be relevant in compromised hosts or revision settings where the risk of surgical site infection is high, but broader comparative evidence remains limited [64,159,160,173].

For fracture-related infection prevention, gentamicin-coated intramedullary nails are a practical example of local antimicrobial delivery on orthopedic hardware, with recent follow-up studies and meta-analyses reporting encouraging infection prevention outcomes in open tibial fracture settings [156,161,162]. In oncologic reconstruction and megaprosthesis surgery, silver-coated implants represent another clinically explored strategy, although their benefit appears to be most relevant in selected high-risk populations and should be weighed against cost, coating durability, and long-term safety considerations [155,163,164].

For DAIR procedures, injectable hydrogels, continuous irrigation systems, bacteriophage delivery, or antibiofilm agents may be more suitable than permanent coatings [25,173]. These systems can be applied after debridement to target residual bacteria in synovial recesses, dead spaces, and periprosthetic soft tissues. For two-stage revision, temporary spacers and degradable carriers may provide local antimicrobial delivery while preserving the reconstructive field. For reimplantation, porous titanium augments, PEEK cages, or bone-contacting implant surfaces may benefit from osteoimmunomodulatory coatings that support late osseointegration.

This application-specific view is important because a material that is unsuitable for an articulating surface may still be highly valuable as a dead-space filler or local delivery platform. Conversely, a mechanically robust alloy may be suitable for load-bearing implants but may not provide the responsive immunomodulatory behavior needed in irregular infected bone defects. Matching material properties to surgical scenario is therefore central to clinical translation [25,28,158].

### 9.6. Toward a Translational Evaluation Framework

To improve clinical readiness, future anti-PJI biomaterials should be evaluated using a structured translational framework [28,158,168]. First, antibacterial performance should be tested against clinically relevant pathogens, including methicillin-susceptible and methicillin-resistant *S. aureus*, *S. epidermidis*, Gram-negative bacteria, fungal pathogens, and polymicrobial biofilms [28,167,168]. Second, biofilm models should include mature EPS-rich biofilms, protein-conditioned implant surfaces, and synovial-like environments. Third, host response should be assessed using immune cell assays, macrophage polarization analysis, neutrophil/ROS responses, osteoclast activity, osteoblast differentiation, angiogenesis, and cytocompatibility [28,153]. Fourth, mechanical testing should be included early [58,158]. Coating adhesion, scratch resistance, press-fit simulation, wear testing, corrosion testing, and fatigue behavior should be matched to the intended anatomical site. Fifth, in vivo models should progress from mechanistic small-animal systems to clinically relevant large-animal models when the material is intended for permanent or load-bearing arthroplasty use. Finally, manufacturing reproducibility, sterilization stability, shelf life, cost and manufacturing scalability, and regulatory classification should be considered before clinical translation [165,171,172].

For more objective comparison across different anti-PJI interface strategies, future studies should report standardized quantitative efficacy metrics whenever possible. These may include bacterial adhesion reduction, viable bacterial count reduction, biofilm biomass reduction, minimum biofilm eradication concentration, antibiofilm penetration depth, antimicrobial release duration, cell viability or cytocompatibility thresholds, osteogenic marker expression, bone–implant contact, pull-out or push-out strength, coating adhesion strength, wear resistance, and ion release parameters under simulated joint loading. Such metrics would allow more direct comparison among anti-adhesive, contact-killing, release-based, matrix-disruptive, infection-responsive, and immunomodulatory strategies.

Together, these criteria emphasize that clinical translation of anti-PJI biomaterials should be assessed beyond isolated antibacterial activity. Promising systems must demonstrate biological efficacy, standardized quantitative performance, host compatibility, mechanical reliability, manufacturing reproducibility, sterilization stability, cost effectiveness, and feasibility in clinically relevant PJI models [25,28,158]. Key translational barriers and clinical readiness considerations are summarized in Table 5.

## 10. Future Perspectives and Conclusions

Anti-PJI biomaterial research is entering a stage in which biological innovation must be matched by clinical feasibility [25,28,64]. To reduce the gap between experimental innovation and clinical application, future development should focus on stage-specific and biofilm-responsive design, immuno-antibacterial osseointegration, and translational validation.

### 10.1. Stage-Specific and Biofilm-Responsive Interface Design

Future prosthetic interfaces should move from static antimicrobial release toward stage-specific regulation [28,55,174]. During the early postoperative or post-revision window, the interface should prevent bacterial adhesion, suppress early biofilm formation, and reduce residual planktonic bacterial burden. After this early infection risk window, cytotoxic antibacterial activity should decrease to permit immune resolution, angiogenesis, osteogenic differentiation, and biological fixation. Sequential systems such as dynamically degrading MgO-modified PEEK illustrate how temporal control may synchronize early antibacterial defense with later osseointegration [121]. In established infection, on-demand activation will be central to precision anti-PJI therapy [25,55]. Future coatings, injectable hydrogels, or local delivery systems should respond to infection-associated cues such as acidic pH, elevated ROS, bacterial enzymes, or biofilm matrix signals [55,158]. Multi-trigger systems may improve specificity by distinguishing true infection from sterile postoperative inflammation while enabling localized release of antimicrobial, antibiofilm, or immunomodulatory agents [55,158].

### 10.2. Immuno-Antibacterial Osseointegration

Future prosthetic interfaces should treat the host immune system as an active therapeutic partner rather than a passive bystander [55]. Instead of suppressing inflammation indiscriminately, immuno-antibacterial interfaces should support early bacterial control and then promote timely transition toward pro-resolving and pro-regenerative immune responses. By modulating neutrophil activity, macrophage phenotype transition, ROS burden, and osteoclastogenesis, such interfaces may interrupt the cycle of chronic inflammation, RANKL-mediated bone resorption, periprosthetic osteolysis, and failed osseointegration [28,55]. This immune regulation should remain stage-specific, avoiding premature immunosuppression during active infection while preventing prolonged inflammatory bone loss after bacterial control.

### 10.3. Artificial Intelligence-Assisted Design and Risk Prediction

Artificial intelligence and machine learning may provide useful tools to accelerate anti-PJI interface research, although their role should be viewed as supportive rather than replacing experimental validation. In antimicrobial discovery, machine learning models could help screen antimicrobial or antibiofilm peptides, predict structure–activity relationships, and prioritize candidates with favorable antibacterial activity, stability, and cytocompatibility [175]. In surface and material design, data-driven approaches may assist in identifying antifouling surfaces, optimizing coating composition, and selecting combinations of antimicrobial agents, matrix-disruptive components, and immunomodulatory cues [176]. These approaches may also support the rational design of multi-stimuli-responsive systems that react to infection-associated changes such as acidic pH, elevated reactive oxygen species, bacterial enzymes, or inflammatory biomarkers. Clinically, artificial intelligence models integrating imaging features, microbiological results, inflammatory markers, comorbidities, and surgical variables may help predict patient-specific PJI risk, support early diagnosis, and guide individualized prevention or revision strategies [177,178]. However, future application will require high-quality multicenter datasets, external validation, interpretability, bias control, and integration with clinically realistic biomaterial testing before routine clinical translation.

### 10.4. Translational Validation and Clinical Readiness

Mechanical durability and standardized evaluation must become core requirements for future anti-PJI interface design. Anti-PJI interfaces intended for arthroplasty should withstand press-fit insertion, cyclic loading, micromotion, synovial lubrication, corrosion, and long-term tribological wear [153,158]. Soft hydrogels or responsive coatings may be suitable for dead-space filling or DAIR adjuncts, whereas permanent load-bearing implants may require durable alloys, PEEK substrates, additive manufacturing, ion implantation, or protected hierarchical architectures [28,58,158].

Future studies should adopt tiered and clinically relevant evaluation systems that integrate mature biofilm models, protein-conditioned implant surfaces, synovial-like environments, polymicrobial and non-staphylococcal pathogen models, immune cell assays, osteogenesis, angiogenesis, cytocompatibility, and mechanical testing [28,167,168]. Preclinical studies should progress from mechanistic small-animal models to large-animal load-bearing PJI models when permanent arthroplasty implants are intended [28]. Manufacturing reproducibility, sterilization compatibility, shelf-life stability, cost effectiveness, regulatory classification, and application-specific surgical workflow should also be considered early in material development [25,36].

### 10.5. Conclusions

In conclusion, the future of PJI management should focus on prosthetic interfaces that integrate microbiology, immunology, bone biology, and mechanical engineering. The central goal is not simply to improve bacterial killing, but to restore a stable prosthetic interface in which biofilms are controlled, inflammation resolves, bone regeneration proceeds, and the implant maintains long-term mechanical durability. Multifunctional immuno-antibacterial osseointegrative interfaces therefore represent a promising direction for achieving durable infection control and biological fixation in arthroplasty.

## Figures and Tables

**Table 1:** The biofilm–immune–bone axis in PJI: pathogenic processes and therapeutic implications.

Pathogenic Axis	Key Pathological Events	Main Therapeutic Target	Interface-Based Strategy
Surface colonization	Protein conditioning film formation, bacterial adhesion, and early microcolony formation	Prevent bacterial attachment and early biofilm initiation	Anti-adhesive coatings, contact-killing surfaces, antimicrobial peptide or metal ion coatings
Mature biofilm barrier	EPS/eDNA matrix formation, antibiotic tolerance, immune shielding, and persister cell survival	Disrupt the biofilm matrix and expose embedded bacteria	DNase/DNase-mimetic systems, EPS-degrading enzymes, phage-based approaches, physical biofilm disruption
Protected microbial reservoirs	Intracellular persistence, abscess-like structures, small-colony variants, and OLCN invasion	Reduce bacterial persistence beyond the visible implant surface	Penetrative local delivery systems, sustained antibiofilm agents, bone-targeted or phage-based therapeutics
Dysregulated immune response	Ineffective neutrophil/macrophage clearance, excessive ROS, chronic inflammation, and immune exhaustion	Restore effective antimicrobial defense while limiting tissue injury	ROS-responsive hydrogels, osteoimmunomodulatory coatings, macrophage-reprogramming interfaces
Periprosthetic osteolysis	Wear debris–bacterial synergy, RANKL-mediated osteoclastogenesis, osteoblast suppression, and bone loss	Inhibit inflammatory bone resorption and protect the residual bone bed	Osteoimmunomodulatory surfaces, Mg/Sr/Cu/Zn-based bioactive interfaces, anti-osteoclastogenic strategies
Failed osseointegration	Poor vascularization, impaired osteogenesis, unstable biological fixation, and recurrent loosening	Restore angiogenesis–osteogenesis coupling and durable bone ingrowth	Porous revision implants, osteogenic/angiogenic coatings, stage-specific immuno-antibacterial interfaces

**Table 2:** Early-stage biofilm-preventive surface engineering strategies for prosthetic joint interfaces.

Strategy	Representative Materials/Designs	Main Anti-Biofilm Mechanism	Key Advantages and Limitations
Anti-adhesive/anti-fouling surfaces	PEGylated coatings; zwitterionic polymers; hydrophilic polymer brushes; slippery liquid-infused surfaces	Reduce nonspecific protein adsorption, modulate conditioning-film composition, and limit early bacterial attachment	Useful for early prevention without relying on antibiotic release; limited activity once bacteria have irreversibly adhered or formed biofilms; may be compromised by synovial protein fouling
Contact-killing surfaces	Quaternary ammonium compounds; cationic polymers; *N*-halamine coatings; antimicrobial peptide-immobilized surfaces	Kill bacteria upon direct contact through electrostatic membrane disruption, membrane permeabilization, or oxidative halogen transfer	Non-systemic and potentially long-acting; efficacy may decline after coverage by dead bacteria, fibrin, inflammatory exudates, or synovial proteins
Mechano-bactericidal nanotopographies	Nanopillars; nanospikes; nanotubes; micro/nano hierarchical titanium surfaces	Mechanically stretch, deform, or rupture bacterial membranes without chemical release	Antibiotic-independent and potentially resistance-sparing; fragile nanostructures may be damaged during press-fit insertion or long-term micromotion
Antibiotic-loaded coatings	Gentamicin, vancomycin, tobramycin, rifampicin, or dual-antibiotic coatings; DAC hydrogel-based local delivery	Provide high local antibiotic concentration at the prosthetic interface during the perioperative contamination window	Clinically intuitive and relatively translatable; burst release, drug depletion, subtherapeutic elution, and resistance selection remain major concerns
Metal ion-based antibacterial coatings	Silver, iodine, copper, zinc, or gallium-containing coatings	Disrupt bacterial membranes, induce oxidative stress, inhibit enzymes, and interfere with bacterial metabolism	Broad-spectrum activity and clinical evidence for Ag/I systems in selected settings; dose-dependent cytotoxicity and long-term ion release must be controlled
Antimicrobial peptide-functionalized coatings	LL-37, HHC36, GL13K, synthetic AMPs, AMP–polymer conjugates	Disrupt bacterial membranes and may modulate immune responses depending on peptide design	Broad antibacterial activity with lower classical resistance risk; limited by proteolytic instability, cost, and uncertain long-term performance
Osteogenic–antibacterial dual-function coatings	CaP/HA plus Ag/Cu/Zn; bioactive glass; Mg-, Sr-, or Zn-doped coatings; osteogenic peptide–antibacterial hybrid coatings	Combine early bacterial inhibition with promotion of osteoblast adhesion, osteogenesis, angiogenesis, or immunocompatible osseointegration	Improves early antibacterial protection while supporting host cell compatibility; release dose, coating adhesion, and mechanical durability remain critical
Load-bearing antibacterial surface integration	Cu-containing CoCrMo alloys; HA-reinforced antibacterial alloys; ion-implanted titanium; durable PEEK or porous titanium modifications	Incorporate antibacterial function into mechanically robust substrates rather than fragile surface coatings	More suitable for high-load arthroplasty than fragile coatings; manufacturing complexity, tribocorrosion, debris safety, and long-term validation remain challenges

Surface engineering strategies for PJI prevention primarily target the earliest stages of infection, including protein conditioning, bacterial adhesion, and early microcolony formation. Although anti-adhesive, contact-killing, antibiotic-releasing, metal ion-based, and dual-function coatings can reduce initial bacterial colonization, their long-term performance depends on coating stability, resistance to synovial fouling, cytocompatibility, and mechanical durability under load-bearing arthroplasty conditions.

**Table 3:** Representative strategies for disrupting mature PJI biofilms.

Strategy	Representative Approaches	Main Anti-Biofilm Mechanism	Key Advantages and Limitations
EPS matrix degradation	Dispersin B; proteases; alginate lyase; polysaccharide- or protein-degrading enzymes	Degrade non-eDNA EPS components, including polysaccharides and proteins; destabilize biofilm architecture and expose embedded bacteria	Directly targets the biofilm barrier; may enhance antibiotic or immune cell penetration; enzyme instability, short activity duration, and local retention remain challenges
eDNA-targeted biofilm destabilization	DNase delivery; DNase-mimetic nanozymes; Ce-based synthetic nuclease mimics; catalytic nanoplatforms	Cleave eDNA scaffolds that stabilize biofilm cohesion, metal ion binding, and horizontal gene transfer	Highly relevant to mature biofilm structure; may reduce matrix-mediated tolerance; catalytic specificity, biosafety, and clearance require validation
Biofilm maturation interference and dispersal	Agr system modulation; quorum-sensing inhibitors; D-amino acids; biofilm-dispersing peptides	Interfere with bacterial communication, biofilm maturation, or cell wall remodeling; promote biofilm disassembly	May prevent maturation or sensitize biofilms to other therapies; efficacy is species- and strain-dependent, and dispersed bacteria require concurrent killing to prevent dissemination
Bacteriophage-based therapy	Personalized phage cocktails; phage-loaded hydrogels; phage-coated implants; depolymerase-producing phages	Specifically infect and lyse target bacteria; some phages degrade biofilm matrix through depolymerases	Useful against multidrug-resistant pathogens; high specificity may preserve host flora; requires pathogen matching, susceptibility testing, and regulatory standardization
Emerging biologic antibiofilm approaches	Anti-biofilm antibodies; matrix-targeting biologics; agents enhancing complement or phagocytosis	Neutralize biofilm-associated structures or promote immune recognition and clearance	Potentially complements antibiotics and surgery; currently limited by cost, tissue penetration, immune reactions, and limited PJI-specific evidence
Photothermal/photodynamic biofilm disruption	Polydopamine, gold nanorods, graphene oxide, black phosphorus, photosensitizer-based systems	Generate local heat or reactive oxygen species (ROS) to damage bacteria and disrupt biofilm integrity	Effective in many preclinical biofilm models; limited by tissue penetration, thermal/oxidative injury risk, and difficulty reaching deep prosthetic joints
Light-assisted chemical antibiofilm therapy	Photodynamic therapy; photothermal therapy; light-assisted chemical approaches	External light activation enhances bacterial killing or biofilm disruption through thermal, photochemical, or oxidative mechanisms	Useful for localized biofilm disruption, but limited by optical penetration, dose control, and host cell safety in deep joints
Magnetic, ultrasound, or electromagnetic activation	Magnetic hyperthermia; sonodynamic therapy; ultrasound-triggered release; electromagnetic induction heating	Provide external energy to disrupt biofilms, enhance drug penetration, or heat metallic implant surfaces	More suitable than light for deep tissues; requires precise dosimetry, device compatibility, and safety control to avoid collateral tissue injury
Combination antibiofilm therapy	EPS degradation + antibiotics; phage + antibiotics; antiseptic combinations; physical activation + immune modulation	Combines matrix disruption, antimicrobial killing or suppression, and improved access to biofilm-associated bacteria	More consistent with mature biofilm complexity, but timing, dosing, safety, and surgical compatibility require optimization

Mature PJI biofilms are protected by EPS-rich matrices containing eDNA, dormant bacterial phenotypes, persister cells, and reduced immune accessibility. Strategies for mature biofilm disruption therefore aim to degrade the biofilm matrix, expose embedded bacteria, enhance antimicrobial penetration, and restore host immune clearance. Because single-modality approaches are unlikely to eradicate chronic PJI biofilms, combination strategies that integrate EPS degradation, local bacterial killing, and immune-compatible local delivery are increasingly favored.

**Table 4:** Smart infection-responsive and osteoimmunomodulatory biomaterials for PJI management.

Strategy	Representative Materials/Triggers	Main Therapeutic Functions	Key Advantages and Limitations
pH-responsive systems	Silk fibroin, chitosan, poly(methacrylic acid), metal–organic frameworks, acid-labile linkers; triggered by acidic infection microenvironment	Release antibacterial agents under acidic conditions; reduce unnecessary release in sterile tissues	Enables on-demand antibacterial delivery; pH heterogeneity and overlap with sterile inflammation may reduce specificity
ROS-responsive and ROS-scavenging systems	Phenylborate ester, thioketal, catechol-containing hydrogels, antioxidant polymer networks; triggered by excessive ROS	Release antimicrobial agents, scavenge excessive ROS, regulate macrophage phenotype, and protect osteogenic cells	Integrates antibacterial therapy with redox and immune regulation; suitable for DAIR adjuncts and dead-space filling, but unsuitable for high-wear articulating or press-fit surfaces
Enzyme-responsive systems	Hyaluronidase-, lipase-, protease-, or nuclease-cleavable carriers; bacterial or inflammation-associated enzyme triggers	Degrade coatings or carriers selectively in infected tissues to release antibiofilm agents	Potentially more infection-specific than pH or ROS alone; enzyme expression varies by pathogen, infection stage, and host response
Multi-stimuli-responsive systems	pH/ROS-responsive hydrogels; pH/enzyme-gated nanoparticles; pH/NIR or ROS/ultrasound dual-responsive systems	Improve specificity by requiring multiple pathological cues for activation	Reduces off-target release and premature activation; design complexity and regulatory burden increase
Gas-releasing platforms	NO-, H_2_S-, or CO-releasing coatings, hydrogels, or nanoparticles	Combine antimicrobial activity with vasoregulation, inflammation control, and tissue-protective effects	Mimics endogenous defense pathways; dose-, flux-, and duration-dependent biological effects require precise release control
External stimulus-responsive systems	Ultrasound-responsive, magnetic hyperthermia, electromagnetic induction heating, sonodynamic or sonothermal systems	Provide spatiotemporal activation, enhance penetration, disrupt biofilms, or trigger local release	Useful for deep prosthetic infections compared with light-based systems; requires device compatibility, precise dosimetry, and safety validation
Stage-specific immune-reprogramming interfaces	Mg^2+^-, Sr^2+^-, Zn^2+^-, Cu^2+^-based coatings; MgO-modified PEEK; sequential degradation or temporally controlled ion release systems	Promote early antimicrobial immune activity followed by pro-resolving, angiogenic, and osteogenic immune responses	Closely matches PJI treatment biology; excessive immune activation or suppression and patient-specific timing remain major challenges
Osteoimmunomodulatory PEEK/titanium interfaces	pH-responsive PEEK; Mg/CNPs/SF coatings; MgO multilayers; micro/nano-structured titanium	Integrate infection-triggered antibacterial activity with immune regulation and osteogenic support	Highly relevant for revision implants and bone-contacting surfaces; requires long-term validation in load-bearing PJI models

Smart anti-PJI biomaterials are designed to convert infection-associated pathological cues, including acidic pH, elevated ROS, bacterial enzymes, and external stimuli, into localized therapeutic activation. Beyond bacterial killing, these systems aim to regulate oxidative stress, macrophage phenotype, angiogenesis, osteogenesis, and immune resolution. Their translational value depends on matching responsive function to anatomical location, mechanical demand, infection stage, and surgical scenario.

**Table 5:** Clinical readiness and translational challenges of current and emerging anti-PJI biomaterial and interface strategies.

Technology/Strategy	Clinical Readiness	Representative Application Scenario	Main Translational Limitations
Systemic antibiotics	High; standard component of prophylaxis and treatment	Perioperative prophylaxis; postoperative systemic therapy; adjunct to DAIR or revision	Limited activity against mature biofilms; toxicity with prolonged use; antimicrobial resistance; poor access to protected microbial reservoirs
DAIR	High; standard option for selected acute PJI	Acute postoperative or acute hematogenous PJI with stable implant and short symptom duration	Timing-dependent efficacy; limited activity against mature biofilms; residual bacteria in modular junctions, synovial recesses, and protected microbial reservoirs
One-stage/two-stage revision surgery	High; widely used for chronic or complex PJI	Chronic PJI, unstable implants, extensive infection, or poor soft-tissue condition	Major surgical morbidity; bone and soft-tissue loss; functional decline; high cost; some patients fail to reach reimplantation
Antibiotic-loaded PMMA spacers/bone cement	High; widely used in staged revision	Two-stage revision; temporary joint spacer; local antibiotic delivery	Burst release followed by low-level elution; nondegradable and biologically inert; limited bone regeneration; potential bacterial recolonization after antibiotic depletion
Antibiotic-loaded calcium sulfate or degradable carriers	Moderate; clinically used in selected settings	Dead-space management; local antibiotic delivery after debridement	Rapid resorption, wound drainage, variable release kinetics, and limited mechanical support
DAC hydrogel and local antibiotic coating systems	Moderate to high; clinical evidence available	Intraoperative implant coating; high-risk arthroplasty, revision procedures, or fracture fixation	Protocol-dependent efficacy; cost; limited long-term comparative data; mainly preventive rather than curative
Silver- or iodine-based antimicrobial implants	Moderate to high; clinical use in selected high-risk settings	Tumor prostheses, compromised hosts, revision reconstruction, or selected high-risk arthroplasty	Cost, coating durability, local toxicity concerns, limited head-to-head comparative evidence, and need for long-term safety data
Gentamicin-coated nails/antibiotic-coated fixation devices	Moderate; strongest evidence in fracture-related infection prevention rather than PJI	Open tibial fractures; high-risk internal fixation; fracture-related infection prevention	Less directly applicable to arthroplasty; indication-specific evidence; resistance risk; coating durability and cost considerations
Anti-adhesive and contact-killing coatings	Preclinical to low clinical readiness; mostly preventive	Primary prevention on non-articulating or bone-contacting implant surfaces	Synovial protein fouling; masking by dead bacteria, fibrin, or inflammatory exudates; coating delamination; limited activity against mature biofilms
Metal ion or antimicrobial peptide coatings	Preclinical to moderate; agent- and indication-dependent	Early colonization prevention; bone-contacting surfaces; high-risk implants	Narrow therapeutic window; dose-dependent cytotoxicity; peptide instability; release control; coating durability; and long-term safety
Smart pH-, ROS-, enzyme-responsive systems	Preclinical/emerging; mostly tested in experimental models	DAIR adjuncts; dead-space fillers; infected bone defects; protected implant surfaces	Trigger specificity; overlap with sterile inflammation; mechanical weakness; sterilization stability; manufacturing scalability; cost; and limited clinical validation
Phage-based or biologic antibiofilm therapy	Preclinical/emerging; experimental and case-based clinical use	Personalized treatment for resistant or recurrent infection; local joint cavity delivery	Pathogen matching; susceptibility testing; immune neutralization; bacterial phage resistance; manufacturing reproducibility; storage, cost, and regulatory complexity
External stimulus-responsive antibiofilm therapy	Preclinical/emerging; mostly preclinical for deep PJI	Photothermal, photodynamic, ultrasound, magnetic hyperthermia, sonodynamic, or electromagnetic activation-assisted therapy	Limited light penetration in deep joints; thermal or oxidative tissue injury; device compatibility; precise dosimetry; and clinical workflow integration
Osteoimmunomodulatory coatings	Preclinical/emerging; strong mechanistic rationale	Revision implants; PEEK or porous titanium bone-contacting surfaces; bone defect repair	Immune complexity, stage-specific timing, long-term fixation, host variability, and limited PJI-specific large-animal data
Load-bearing integrated antibacterial alloys	Preclinical/emerging	Articulating or high-load prosthetic components; durable infection-resistant alloys	Manufacturing complexity, cost and scalability, tribocorrosion, debris safety, regulatory testing, and long-term clinical validation

Current anti-PJI technologies differ substantially in clinical readiness, and translation of emerging systems will require application-specific validation of antimicrobial efficacy, standardized quantitative performance metrics, cytocompatibility, mechanical durability, manufacturing reproducibility, scalability, cost effectiveness, sterilization compatibility, and performance in clinically relevant PJI models.

## Data Availability

No new data were created or analyzed in this study. Data sharing is not applicable to this article.

## Abbreviations

The following abbreviations are used in this manuscript:

TJA	Total joint arthroplasty
PJI	Periprosthetic joint infection
PMMA	Polymethylmethacrylate
EPS	Extracellular polymeric substance
eDNA	Extracellular DNA
OLCN	Osteocyte lacuno-canalicular network
DAIR	Debridement, antibiotics, and implant retention
ALBC	Antibiotic-loaded bone cement
MSCRAMMs	Microbial surface components recognizing adhesive matrix molecules
MRSA	Methicillin-resistant *Staphylococcus aureus*
UHMWPE	Ultra-high-molecular-weight polyethylene
ROS	Reactive oxygen species
NETs	Neutrophil extracellular traps
PAMPs	Pathogen-associated molecular patterns
RANKL	Receptor activator of nuclear factor-κB ligand
OPG	Osteoprotegerin
PEG	Polyethylene glycol
QACs	Quaternary ammonium compounds
AMP	Antimicrobial peptide
Ag	Silver
Cu	Copper
Zn	Zinc
Sr	Strontium
HA	Hydroxyapatite
CaP	Calcium phosphate
CoCrMo	Cobalt–chromium–molybdenum alloy
PEEK	Polyetheretherketone
PDA	Polydopamine
SF	Silk fibroin
DNase	Deoxyribonuclease
NIR	Near-infrared
PTT	Photothermal therapy
PDT	Photodynamic therapy
NO	Nitric oxide
HIF-1α	Hypoxia-inducible factor-1α
VEGF	Vascular endothelial growth factor
BMP-2	Bone morphogenetic protein-2
NF-κB	Nuclear factor-κB
IL-1β	Interleukin-1β
IL-6	Interleukin-6
IL-10	Interleukin-10
TNF-α	Tumor necrosis factor-α
BMSC	Bone marrow mesenchymal stem cell
CFU	Colony-forming unit
AgNPs	Silver nanoparticles
TA	Tannic acid
